# Serum liver enzymes and risk of stroke: Systematic review with meta‐analyses and Mendelian randomization studies

**DOI:** 10.1111/ene.16506

**Published:** 2024-10-10

**Authors:** Chun Li, Long Gu, Fu‐Yi Shi, Shi‐Ying Xiong, Gui‐Sheng Wu, Jian‐Hua Peng, Ruo‐Lan Wang, Yuan Yuan, Yong Jiang, Chen Huang, Huai‐Rong Luo

**Affiliations:** ^1^ State Key Laboratory of Quality Research in Chinese Medicine Macau University of Science and Technology Taipa Macao China; ^2^ Clinical Trial Research Center The Affiliated Traditional Chinese Medicine Hospital, Southwest Medical University Luzhou Sichuan China; ^3^ Laboratory of Neurological Diseases and Brain Function The Affiliated Hospital, Southwest Medical University Luzhou Sichuan China; ^4^ Key Laboratory of Luzhou City for Aging Medicine, Department of Pharmacology School of Pharmacy, Southwest Medical University Luzhou Sichuan China; ^5^ Central Nervous System Drug Key Laboratory of Sichuan Province Luzhou Sichuan China; ^6^ Department of Neurosurgery The Affiliated Hospital, Southwest Medical University Luzhou Sichuan China

**Keywords:** alanine transaminase, alkaline phosphatase, aspartate aminotransferase, gamma‐glutamyltransferase, ischemic stroke

## Abstract

**Background and purpose:**

Previous observational studies have identified correlations between liver enzyme levels and stroke risk. However, the strength and consistency of these associations vary. To comprehensively evaluate the relationship between liver enzymes and stroke risk, we conducted meta‐analyses complemented by Mendelian randomization (MR) analyses.

**Methods:**

Following the PRISMA guidelines, we performed meta‐analyses of prospective studies and conducted subgroup analyses stratified by sex and stroke subtype. Subsequently, adhering to the STROBE‐MR guidelines, we performed two‐sample bidirectional univariable MR (UVMR) and multivariable MR (MVMR) analyses using the largest genome‐wide association studies summary data. Finally, the single‐nucleotide polymorphisms associated with liver enzymes on sex differences underwent gene annotation, gene set enrichment, and tissue enrichment analyses.

**Results:**

In the meta‐analyses of 17 prospective studies, we found the relative risks for serum γ‐glutamyl transferase (GGT) and alkaline phosphatase (ALP) were 1.23 (95% CI: 1.16–1.31) and 1.3 (95% CI: 1.19–1.43), respectively. Subgroup analyses revealed sex and stroke subtype differences in liver enzyme‐related stroke risk. Bidirectional UVMR analyses confirmed that elevated GGT, alanine aminotransferase, and aspartate aminotransferase levels were associated with increased stroke occurrence. The primary results from the MVMR analyses revealed that higher ALP levels significantly increased the risk of stroke and ischemic stroke. Gene set and tissue enrichment analyses supported genetic differences in liver enzymes across sexes.

**Conclusions:**

Our study provides evidence linking liver enzyme levels to stroke risk, suggesting liver enzymes as potential biomarkers for early identification of high‐risk individuals. Personalized, sex‐specific interventions targeting liver enzymes could offer new strategies for stroke prevention.

## INTRODUCTION

Stroke is a prominent contributor to early mortality and enduring incapacitation on a global scale [1]. The fatalities, cognitive decline, and impairments induced by stroke worldwide obviously diminish life quality and burden economies and societies, rendering it a substantial public health concern [2]. The increasing burden and costs associated with stroke management underscore the urgent need for effective stroke prevention strategies. It is noteworthy that over 90% of stroke cases arise from potentially modifiable risk factors, with more than 75% of this burden being potentially reducible through the management of metabolic and behavioral risk factors [3].

Serum liver enzymes, encompassing γ‐glutamyl transferase (GGT), alkaline phosphatase (ALP), alanine aminotransferase (ALT), and aspartate aminotransferase (AST), are commonly utilized as clinical indicators for evaluating liver function. However, these enzymes are not exclusively indicative of liver pathology [4]. Isoenzyme GGT1 primarily participates in the metabolism of glutathione and the regulation of redox balance, while GGT5 is expressed in various tissues and additionally plays a crucial role in inflammatory responses through the conversion of leukotrienes [5]. ALP is differentiated into placental, intestinal, germ cell, and tissue‐nonspecific isoenzymes based on tissue types, with tissue‐nonspecific ALP essential for bone mineralization and neuronal differentiation [6]. ALT, or glutamate‐pyruvate transaminase (GPT), exists as GPT1 in serum and GPT2 in mitochondria [7]. AST, known as glutamate‐oxaloacetate transaminase (GOT), is widely distributed and functions in both the serum (GOT1) and mitochondria (GOT2) [7]. Therefore, serum liver enzyme levels may indicate broader multi‐tissue biological processes. Multiple studies have found associations between liver enzyme levels and the risk of stroke; nonetheless, the results remain controversial and inconclusive [8, 9].

In observational studies, inevitable confounding factors affect the causal inference of liver enzyme levels in stroke risk. Consequently, genetic analyses independent of external factors and disease progression might mitigate confounding effects and reverse causality. Mendelian randomization (MR), an epidemiological method, aids in establishing causal relationships in exposure–outcome associations by employing genetic variation as instrumental variables for exposure [10]. Here, we conducted meta‐analyses to summarize existing evidence from traditional observational studies and performed two‐sample bidirectional univariable Mendelian randomization (UVMR) and multivariable Mendelian randomization (MVMR) analyses to investigate the association between four liver enzyme levels and stroke risk. Our aim was to provide a comprehensive evaluation of these associations, thereby contributing to the foundation for stroke prevention strategies.

## METHODS

### Study registration and methodological compliance

**FIGURE 1:**
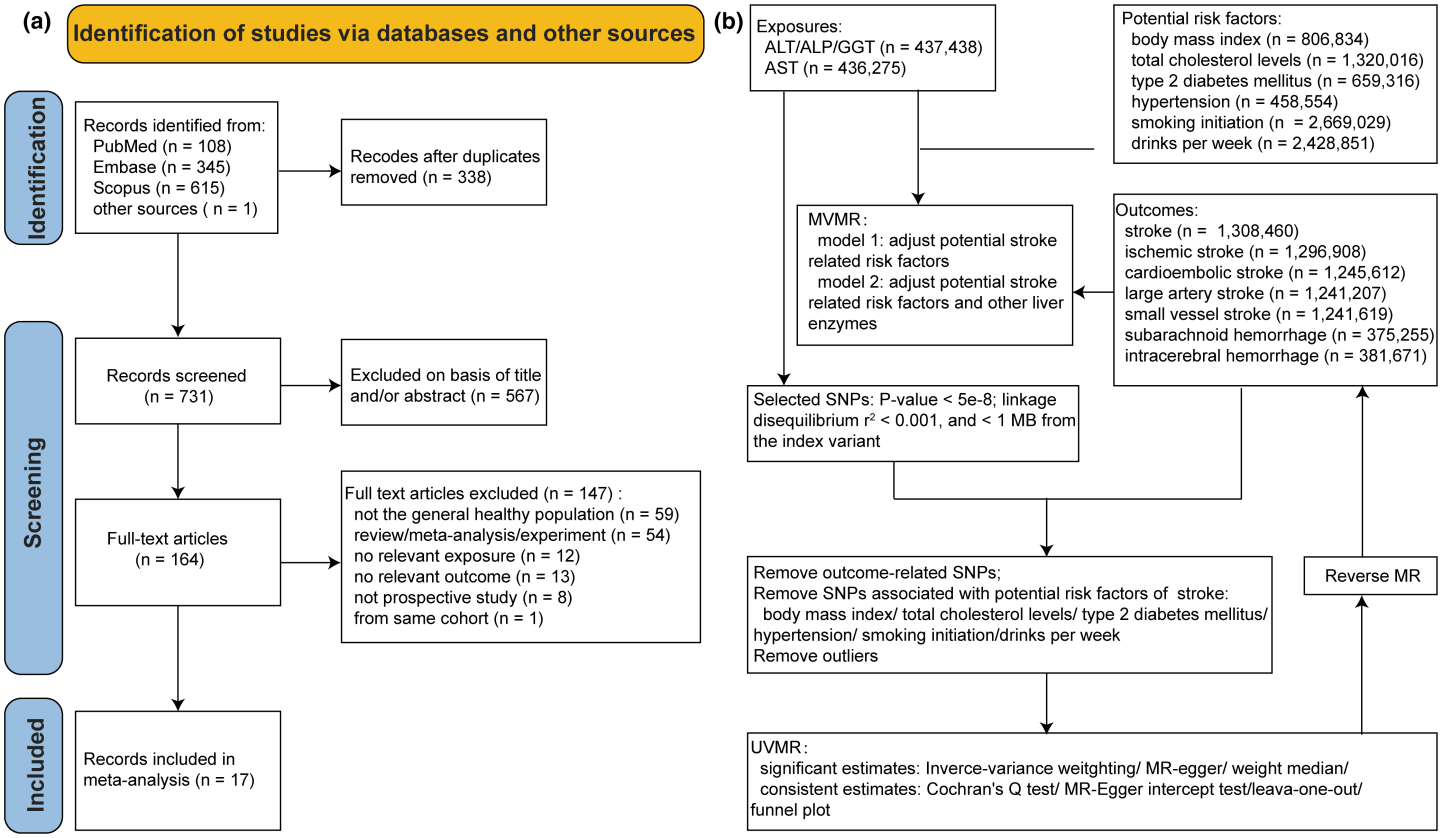
PRISMA flow diagram for study selection for the systematic review (a) and workflow for Mendelian randomization analyses (b). ALP, alkaline phosphatase; ALT, alanine aminotransferase; AST, aspartate aminotransferase; GGT, γ‐glutamyl transferase; MR, Mendelian randomization; MVMR, multivariable Mendelian randomization; PRISMA, Preferred Reporting Items for Systematic Reviews and Meta‐Analyses; SNP, single‐nucleotide polymorphism; UVMR, univariable Mendelian randomization.

The meta‐analyses conducted in this study were registered under PROSPERO (ID: CRD42024525733), and strictly followed the Preferred Reporting Items for Systematic Reviews and Meta‐Analyses (PRISMA) [11] guidelines (Supporting Information File 1). The MR methods were compliant with the STROBE‐MR checklist [12]; further details can be found in Supporting Information File 2. Figure 1 illustrates the PRISMA flow diagram and MR analyses workflow.

### Meta‐analyses of observational studies

We searched electronic databases (PubMed, Embase, and Scopus) for prospective studies, cohort studies, follow‐up studies, and real‐world studies from inception to February 26, 2024. The search strategy included a combination of MeSH (Medical Subject Headings) terms and free‐text terms, as well as a comprehensive set of keywords related to exposures (e.g., “gamma‐glutamyl transferase,” “alkaline phosphatase,” “alanine aminotransferase,” “aspartate aminotransferase,” etc.) and outcome (stroke). We also manually checked reference lists to identify additional potential studies and restricted the search to studies conducted on humans. There were no language or publication date restrictions. Detailed records of the search conducted for all databases are listed in the Supporting Information File 3.

Studies meeting the following criteria were included: (i) baseline levels of liver enzymes (GGT, ALP, ALT, AST) were measured in a general adult population, encompassing both individuals with normal and slightly elevated enzyme levels; (ii) a follow‐up period of at least 1 year; (iii) provision of effect size for stroke outcomes; and (iv) exclusion of participants with a history of stroke or liver disease. Two researchers (C.L. and L.G.) independently screened the titles and abstracts of the literature and obtained the full‐text articles of potentially relevant studies. The quality of the included studies was evaluated using the Newcastle‐Ottawa Scale [13], assigning each study a “star rating.” Quality was classified as good (≥7 stars), fair (4–6 stars), or poor (<4 stars). The extracted data comprised the first author's surname, publication year, duration of follow‐up, number of participants, demographic characteristics (age, sex, body mass index [BMI], smoking and drinking status, population), metabolic parameters (blood glucose, blood pressure, lipid profile, liver enzymes), number of stroke cases, stroke subtypes and incidence, as well as adjusted relative risks (RRs) and 95% confidence intervals (CIs), and adjusted risk factors. For odds ratios (ORs), the data was transformed into RR using the formula: RR = OR/([1–pRef] + [pRef × OR]), where pRef is the prevalence of the outcome in the reference group [14]. Any discrepancies were resolved by consensus of a third author (F.‐Y.S.).

After data extraction, subsequent meta‐analyses were conducted by stratifying based on four specific liver enzymes. Given the clinical and methodological variations among the included studies, such as variances in baseline characteristics of subjects, duration of follow‐up, and adjustment for confounding factors, the DerSimonian and Laird random‐effects model was employed to present the findings, regardless of the presence of heterogeneity [15]. In instances where heterogeneity is absent in the pooled data, the results of both the random‐effects and fixed‐effects models are similar; nevertheless, if substantial heterogeneity is present, the random‐effects model is deemed more conservative [14]. To validate the findings further, both fixed and random‐effects models were applied in forest plots. Heterogeneity among studies was assessed using Cochran's *Q* test and the *I* ^*2*^ statistic. A *P*_*Q*‐statistic_ ≥0.10 and *I* ^*2*^ <50% indicated nonsignificant heterogeneity among the included studies [16]. Subgroup analyses were conducted based on sex, stroke subtype, and the interaction between stroke subtype and sex. We also conducted subgroup comparisons between different populations, where data were available. Publication bias was evaluated via funnel plots alongside Egger and Begg tests [14]. Sensitivity analyses were conducted to evaluate the influence of individual studies on the estimated RR. The analyses were performed using Stata (version 15.0).

### MR analyses

Summary data for serum levels of three liver enzymes (ALT, ALP, GGT) were collected from genome‐wide association studies (GWASs) including 437,438 individuals of European ancestry [17]. Serum AST data was obtained from the GWAS data including 436,275 individuals of European ancestry from the UK Biobank (UKB) [18]. GWAS summary data related to stroke were acquired from the GIGASTROKE consortium, including 73,652 stroke cases and 1,234,808 controls of European ancestry [19]. The GIGASTROKE study encompasses 62,100 cases of ischemic stroke (IS), systematically identified according to the Trial of ORG 10172 in Acute Stroke Treatment (TOAST) criteria, and three distinct IS subtypes were classified: large vessel atherosclerosis stroke (LVS, *n* = 6399), small vessel occlusion stroke (SVS, *n* = 6811), and cardioembolic stroke (CES, *n* = 10,804). GWAS data for subarachnoid hemorrhage (SAH) and intracerebral hemorrhage (ICH) were derived from FinnGen [20], with SAH comprising 3532 cases and 371,753 controls, and ICH comprising 7040 cases and 374,631 controls. Additionally, GWAS summary data for total cholesterol levels (TC), hypertension (HT), smoking initiation (SI), drinks per week (DPW), type 2 diabetes mellitus (T2DM), and BMI were obtained for adjusting confounding factors [21, 22, 23, 24, 25].

We rigorously uphold the three key assumptions: relevance, exchangeability, and exclusion restriction principle, during the execution of our MR analyses. Single‐nucleotide polymorphisms (SNPs) closely associated with serum enzyme activity of individual liver enzymes were identified using a significance threshold of 5e‐8. This threshold was chosen to ensure strong instrument relevance while minimizing the risk of false‐positives, a standard practice in large‐scale GWAS‐based MR analyses [19]. Linkage disequilibrium (LD) among SNPs was alleviated (*r* ^2^ < 0.001, kb = 1000) to ensure the independence of instrumental variables. Each SNP was examined using the online tools LDtrait [26] and Ensembl [27] to evaluate its correlation with confounding variables. This additional step was critical to ensure that the selected SNPs were not associated with potential confounders, thus addressing the exchangeability assumption in MR analysis. SNPs related to confounding factors were removed, and MR‐PRESSO was used for outlier detection (*p* < 0.05) [28]. After estimating the phenotypic variance explained by instrumental variables (*R* ^2^) and calculating the *F*‐statistics to assess their strength, we conducted UVMR analyses following the removal of weak instrument bias (*F*‐value <10) for SNPs [29].

To address potential pleiotropy, we employed several sensitivity analyses, including tests for heterogeneity, MR‐Egger regression, and the weighted median method. These methods are specifically designed to detect and adjust for pleiotropy, ensuring that any observed associations are not driven by pleiotropic effects of the SNPs. MR‐Egger, for example, can test for directional pleiotropy, while the weighted median method can provide reliable estimates even when up to 50% of the SNPs are invalid. Additionally, we performed pleiotropy tests, constructed funnel plots, and conducted leave‐one‐out analyses to ensure the fulfillment of the key assumptions of MR. These steps allowed us to further confirm the robustness of our results, accounting for any potential violations of the MR assumptions. In the reverse MR analyses, a screening threshold of *p* < 1 × 10^−5^ was applied due to limited SNP availability.

Since MR assesses the “total” effect of exposure on the outcome, while MVMR evaluates the “direct” effect of each exposure on the outcome [30], we utilized MVMR analyses to further evaluate the causal relationship between each liver enzyme and stroke. To alleviate markedly multicollinearity issues resulting from excessive exposure variables, each liver enzyme underwent bidirectional UVMR analyses, utilizing common adjustment factors from observational studies including TC, HT, SI, DPW, T2DM, and BMI. The significant adjustment factors identified were subsequently incorporated as covariates in MVMR analyses model 1. This approach ensured that key confounding variables, known to influence both liver enzyme levels and stroke risk, were appropriately accounted for. To mitigate the mutual influence among liver enzymes, we performed model 2 MVMR analyses, which included additional adjustment for the other three liver enzymes. By adjusting for the other liver enzymes in model 2, we aimed to isolate the independent effects of each enzyme on stroke risk, thus strengthening the validity of our findings.

Analyses were performed using R packages TwoSampleMR (version 0.5.8) [31], MR‐PRESSO (version 1.0), and LDlinkR (version 1.3.0) [26] in R version 4.2.3. A *P*‐threshold of 0.05/3 (number of stroke subtypes or IS subtypes) was defined as statistical significance.

### Enrichment analyses

FUMA was employed to conduct gene annotation and tissue enrichment analyses for significant SNPs (*p* < 5e‐8) associated with liver enzymes based on sex differences [32]. Furthermore, gene set enrichment analyses were carried out using clusterProfiler (version 4.5.1) [33].

## RESULTS

### Meta‐analyses of observational studies

The study retrieved 1069 publications from electronic databases and other sources. After evaluating these studies and their referenced reviews, we included 17 studies investigating the correlation between serum GGT (*n* = 13), ALP (*n* = 5), ALT (*n* = 6), and AST (*n* = 4) levels and stroke risk (Supporting Information File 3). These studies involved a combined population of 2,365,837 individuals, with 35,484 stroke patients and 2,330,353 controls. Detailed information for each study can be found in Table 1. The average Newcastle‐Ottawa Scale score was 8.3 (range: 6–9), suggesting a relatively high quality of the included studies, as described in Table S1.

**TABLE 1:** Characteristics of included studies in meta‐analyses.

Study	Location	Age range at baseline (years)	Mean follow‐up (years)	Exposure	Participants (male/female) (*n*)	Events (male/female) (*n*)	Study quality
Jousilahti 2000	Finland	25–64	7	GGT	14,874 (7176/7698)	470 (261/209)	8
Bots 2002 (UK)	UK	45–59	11–15^a^	GGT	181 (181/0)	57 (57/0)	7
Bots 2002 (Fin)	Finland	42–60	3–9^a^	GGT	200 (200/0)	66 (66/0)	7
Bots 2002 (The Net)	Netherlands	≥55	1–4^a^	GGT	429 (152/277)	108	6
Kim 2005	Korea	35–59	10	ALT; AST	108,464 (108,464/0)	3022 (3022/0)	8
Ebrahim 2006	Korea	30–64	15	GGT	500,419	3133	8
Fraser 2007	UK	60–79	4.6	GGT; ALT	2961 (0/2961)	40 (0/40)	8
Wannamethee 2008	UK	40–59	24	GGT	6997 (6997/0)	549 (549/0)	8
Shimizu 2010	Japan	40–69	18.1	GGT	9752 (3471/6281)	432 (230/202)	9
Wieberdink 2011	Netherlands	≥55	13.9	ALP	4876	716	8
Weikert 2013	Germany	35–65	8.2	GGT; ALT	2419	353	9
Shimizu 2013	Japan	40–69	16	ALP	10,754 (4098/6656)	489 (264/225)	9
Wannamethee 2013	UK	60–79	11	ALP	3381 (3381/0)	230 (230/0)	8
Kabootari 2018	Iran	≥30	11.3	ALP	2578 (1089/1489)	68	9
Yang 2020	Korea	40–69	12	GGT; ALT; AST	7964 (3312/4652)	168	8
Wang 2022	China	30–79	10	GGT; ALT; AST	15,686 (7959/7727)	9128 (4439/4689)	9
Baek 2023	Korea	>40	6.4	GGT	1,640,127 (1,195,230/444,897)	14,563	9
Arafa 2023	Japan	30–79	16.7	GGT; ALT; AST	7386 (3379/4007)	438 (234/204)	9
Liu 2023	China	Male: 65.2 (6.5); Female: 58.9 (8.0)^b^	7.3	ALP	26,389 (11,408/14,981)	1454 (913/541)	9

Abbreviations: ALP, alkaline phosphatase; ALT, alanine aminotransferase; AST, aspartate aminotransferase; GGT, γ‐glutamyl transferase.

^a^ Range of follow‐up.

^b^ Mean (standard deviation) age at baseline.

**FIGURE 2:**
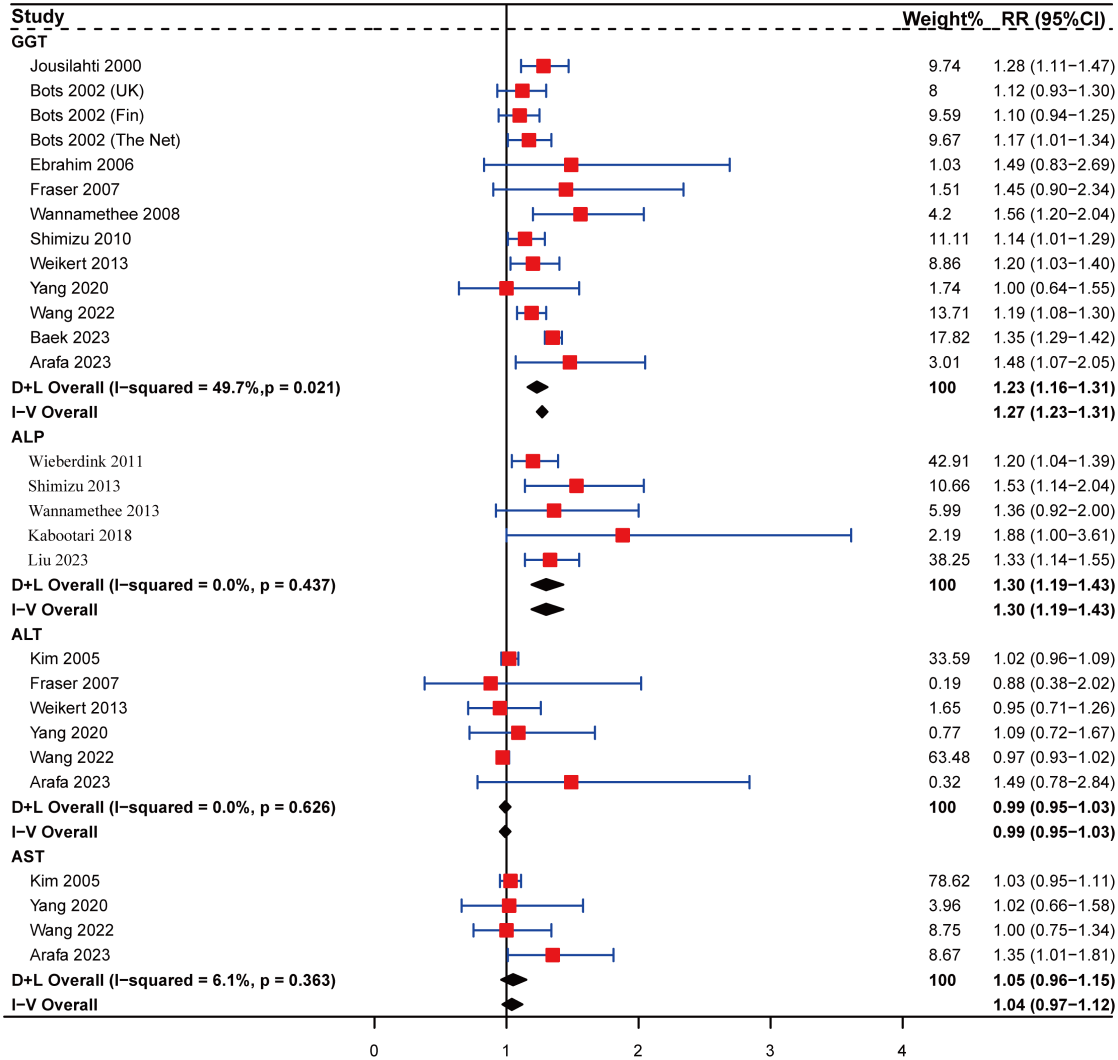
Forest plot for the associations of serum γ‐glutamyl transferase (*n* = 13), alkaline phosphatase (*n* = 5), alanine aminotransferase (*n* = 6), and aspartate aminotransferase (*n* = 4) with risk of stroke. Squares represent the estimate of relative risk for each study; the horizontal lines represent the 95% confidence interval (95% CI), and diamonds represent the overall estimate and its 95% CI. ALP, alkaline phosphatase; ALT, alanine aminotransferase; AST, aspartate aminotransferase; CI, confidence interval; D + L, DerSimonian and Laird random effects model; GGT, γ‐glutamyl transferase; I‐V, inverse variance fixed effects model; RR, relative risk.

The meta‐analyses revealed a significant association between elevated serum GGT and ALP levels and an increased risk of stroke, whereas heightened ALT and AST levels showed no significant alteration in stroke risk (Figure 2). Compared with the low GGT level group, the high GGT level group had a 23% increased risk of stroke (RR: 1.23, 95% CI: 1.16–1.31). Likewise, individuals with elevated serum ALP levels had a 30% increased risk of stroke (RR: 1.3, 95% CI: 1.19–1.43) compared with those with lower serum ALP levels. Sensitivity analyses revealed that all studies, except for Baek 2023 in the GGT group, passed sensitivity testing, as shown in Figure S1. However, after excluding the study by Baek et al., the result of meta‐analysis did not reveal any changes in directional consistency or significance of the overall effect (RR: 1.19, 95% CI: 1.14–1.25; Figure S2).

**FIGURE 3:**
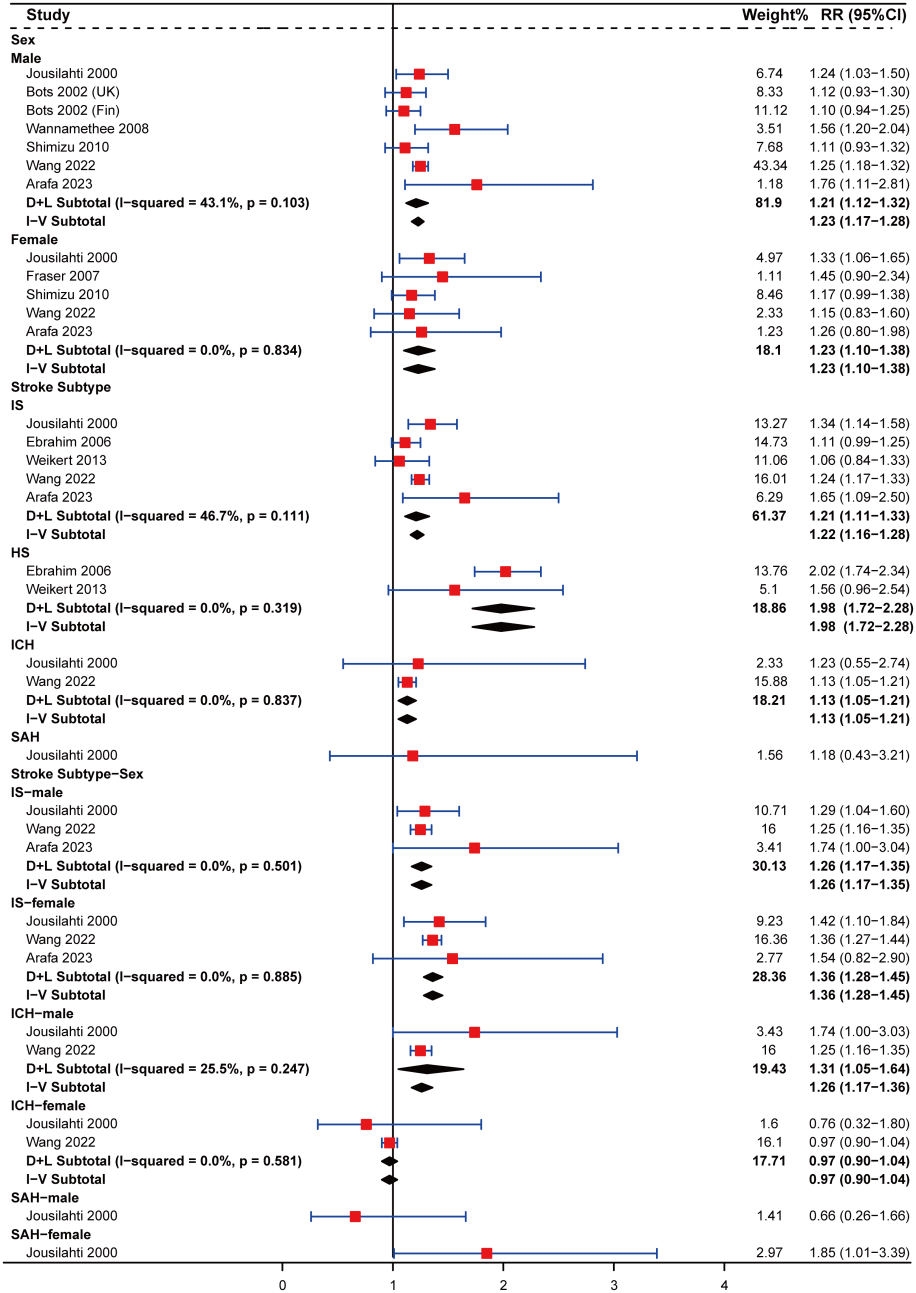
Subgroup analyses on the associations of serum γ‐glutamyl transferase level with stroke stratified by sex, stroke subtype, and stroke subtype‐sex. Squares represent the estimate of relative risk for each study; the horizontal lines represent the 95% confidence interval (95% CI), and the diamonds represent the overall estimate and its 95% CI. CI, confidence interval; D + L, DerSimonian and Laird random effects model; GGT, γ‐glutamyl transferase; HS, hemorrhagic stroke; ICH, intracerebral hemorrhage; IS, ischemic stroke; I‐V, inverse variance fixed effects model; RR, relative risk; SAH, subarachnoid hemorrhage.

In the subgroup analyses related to GGT, no heterogeneity was observed within any subgroup based on sex or stroke subtype (Figure 3). Thus, it is postulated that the heterogeneity observed in the total meta‐analysis associated with GGT (Figure 2) may be attributed to sex or stroke subtype. As shown in Figure 3, the RR (95% CI) for IS, ICH, and hemorrhagic stroke (HS) in individuals with high GGT levels were 1.21 (1.11–1.33), 1.13 (1.05–1.21), and 1.98 (1.72–2.28), respectively. Males with high baseline GGT levels had an increased risk of IS and ICH, with RRs (95% CI) of 1.26 (1.17–1.35) and 1.31 (1.05–1.64), respectively. Conversely, among females, the risk of IS increased (RR: 1.36, 95% CI: 1.28–1.45) without an increased risk of ICH (RR: 0.97, 95% CI: 0.90–1.04).

**FIGURE 4:**
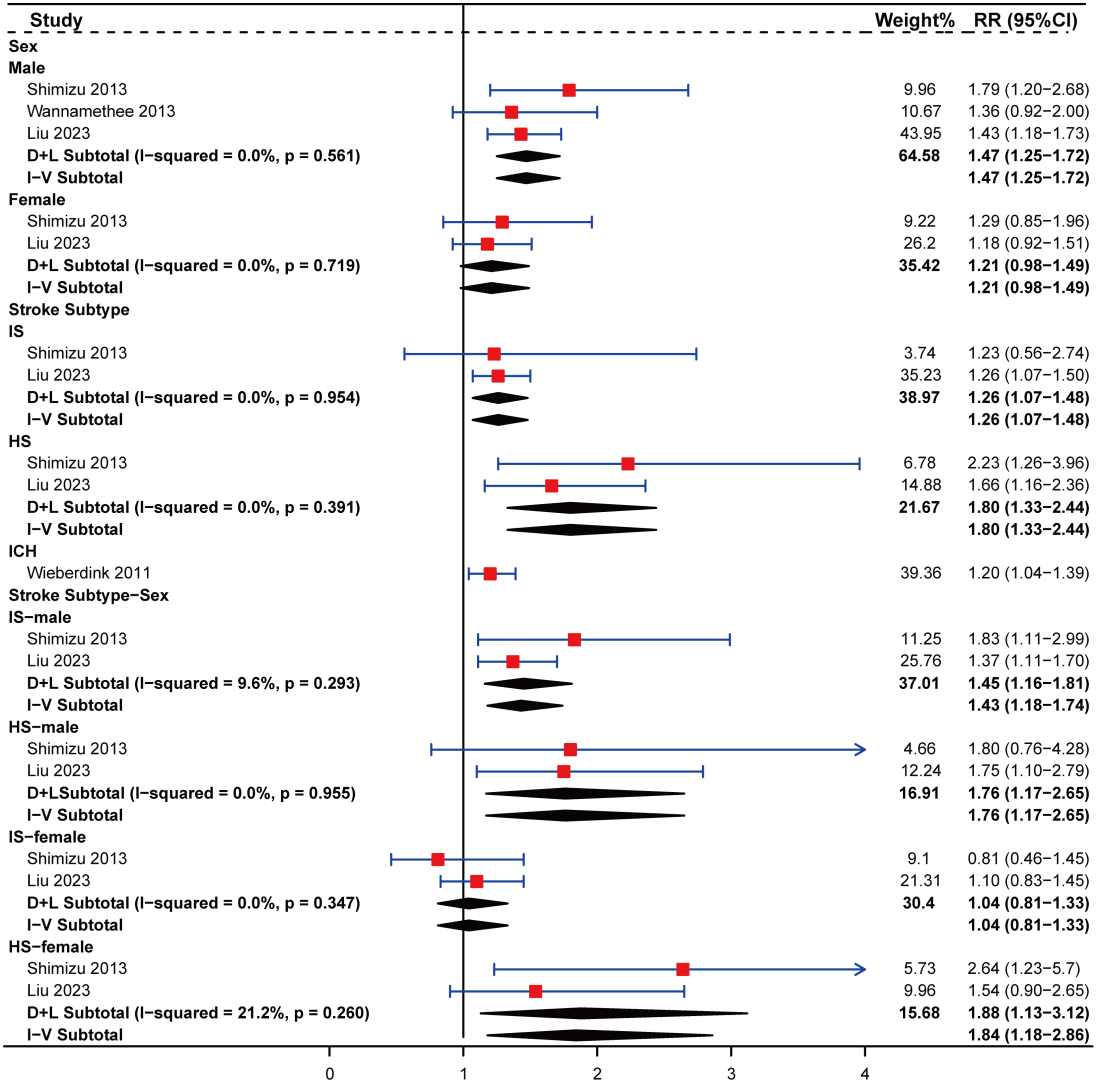
Subgroup analyses on the associations of serum alkaline phosphatase level with stroke stratified by sex, stroke subtype, and stroke subtype‐sex. Squares represent the estimate of relative risk for each study; the horizontal lines represent the 95% confidence interval (95% CI), and the diamonds represent the overall estimate and its 95% CI. ALP, alkaline phosphatase; CI, confidence interval; D + L, DerSimonian and Laird random effects model; HS, hemorrhagic stroke; ICH, intracerebral hemorrhage; IS, ischemic stroke; I‐V, inverse variance fixed effects model; RR, relative risk.

In the subgroup analyses concerning ALP, significant differences in stroke risk linked to elevated ALP levels were apparent across sexes (Figure 4). Stroke risk was higher in males, with an RR (95% CI) of 1.47 (1.25–1.72), whereas females did not exhibit an elevated stroke risk, with an RR (95% CI) of 1.21 (0.98–1.49). Subgroup analysis based on stroke subtype revealed that high ALP levels were associated with an increased risk of IS and HS in the general population, with RRs (95% CI) of 1.26 (1.07–1.48) and 1.80 (1.33–2.44), respectively. Additional analysis based on stroke subtype and sex revealed that heightened ALP levels correlated with a higher risk of both IS and HS in males, with RRs (95% CI) of 1.45 (1.16–1.81) and 1.76 (1.17–2.65). However, females displayed an increased risk of HS, indicated by an RR (95% CI) of 1.88 (1.13–3.12), while not experiencing a corresponding increase in IS risk (RR: 1.04, 95% CI: 0.81–1.33).

In subgroup analyses of ALT, we found no association between elevated ALT levels and stroke risk across sexes or stroke subtypes (Figure S3a,b). However, in sex‐stratified subgroup analysis of ALT for stroke subtype, elevated ALT levels were associated with an increased risk of ICH in males (Figure S3c), with an RR (95% CI) of 1.07 (1.05–1.09). In subgroup analyses of AST, no significant association was found between elevated enzyme levels and stroke risk on sex differences (Figure S4a). However, elevated AST levels were associated with a higher risk of ICH (RR: 1.12, 95% CI: 1.05–1.18; Figure S4b), exclusively observed in males (RR: 1.09, 95% CI: 1.07–1.11; Figure S4c). Examination of the symmetry of the funnel plot and the results of Egger and Begg tests provided assurance of the absence of publication bias in the aforementioned results (Figure S5). Moreover, subgroup analyses of stroke risk among different ancestral populations based on GGT and ALP did not reveal any significant differences in their impact on stroke risk (Figure S6).

### MR analyses

#### Bidirectional UVMR analyses

The sources and relevant information of the GWAS data are provided in Table S2. Following the initial screening, 251 independent SNPs related to ALT, 405 to ALP, 356 to GGT, and 264 to AST were identified. We estimated the phenotypic variance explained by *R* ^2^ and calculated the *F*‐statistics to assess the strength of instrumental variables (Table S3). Subsequently, we used LDtrait and Ensembl to exclude 284 SNPs associated with potential risk factors (Table S4). Furthermore, we employed MR‐PRESSO to remove six outliers (Table S5) and harmonized outcome SNPs before conducting the two‐sample MR analyses. A causal inference was inferred when an effect estimate achieved statistical significance in the inverse variance weighted analysis and maintained directional consistency across the other analytical approaches.

**FIGURE 5:**
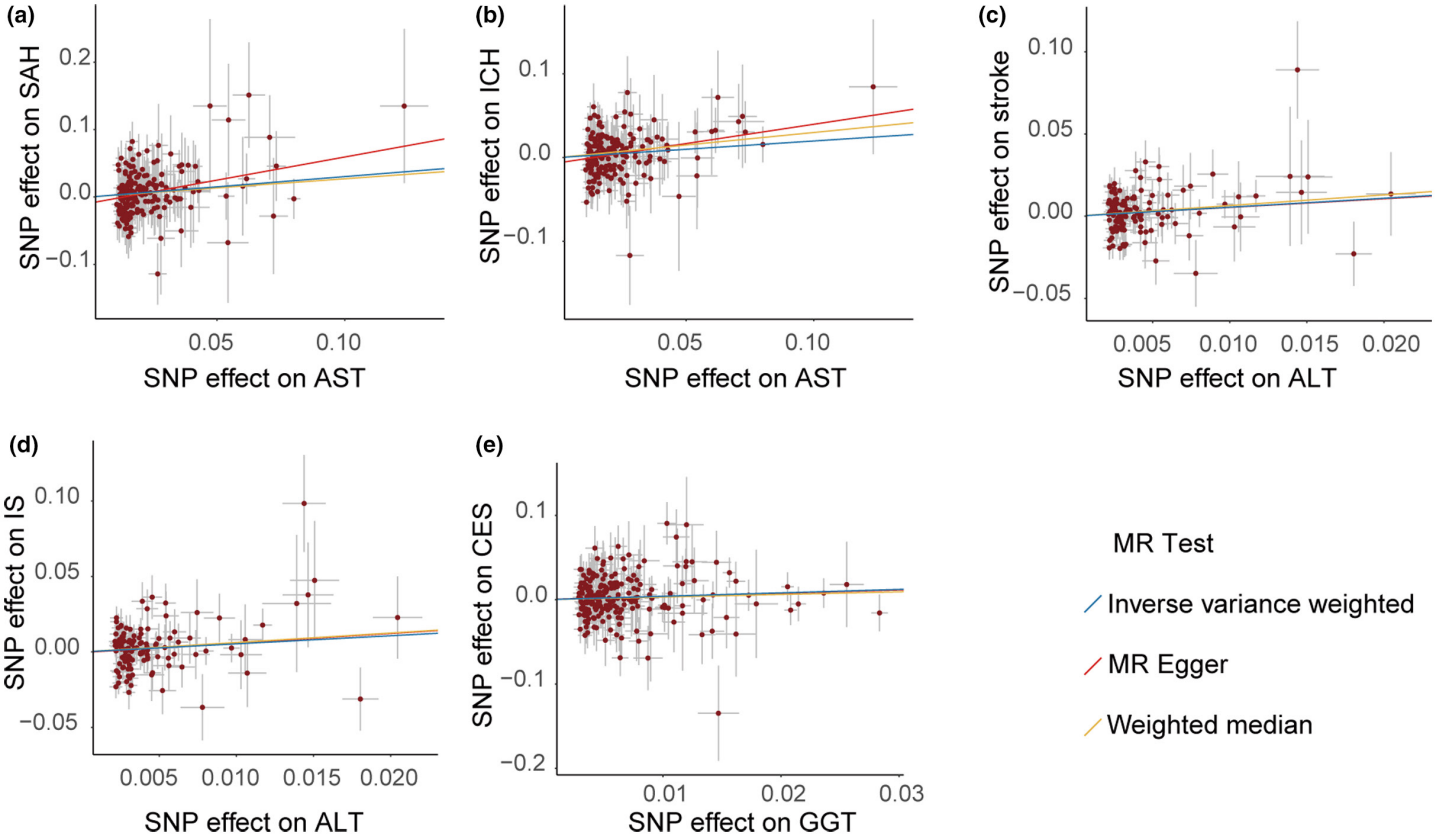
Scatterplots assess the effects of aspartate aminotransferase on subarachnoid hemorrhage (a) and intracerebral hemorrhage (b), the effect of alanine aminotransferase on stroke (c) and ischemic stroke (d), and the effect of γ‐glutamyl transferase on cardioembolic stroke (e). ALT, alanine aminotransferase; AST, aspartate aminotransferase; CES, cardioembolic stroke; GGT, γ‐glutamyl transferase; ICH, intracerebral hemorrhage; IS, ischemic stroke; MR, Mendelian randomization; SAH, subarachnoid hemorrhage; SNP, single nucleotide polymorphism.

We observed that AST activity levels exhibited a statistical significant causal effect on SAH (Figure 5a, nSNPs = 189, OR: 1.35, 95% CI: 1.12–1.64, *p* = 0.002) and ICH (Figure 5b, nSNPs = 189, OR: 1.22, 95% CI: 1.06–1.40, *p* = 0.005). ALT showed nominal significant associations with stroke (Figure 5c, nSNPs = 130, OR: 1.73, 95% CI: 1.10–2.70, *p* = 0.017) and IS (Figure 5d, nSNPs = 131, OR: 1.71, 95% CI: 1.03–2.84, *p* = 0.038). Additionally, the results revealed a nominal significant causal relationship between GGT and CES risk (Figure 5e, nSNPs = 227, OR: 1.50, 95% CI: 1.03–2.18, *p* = 0.033). No nominal significant causal associations were observed between other outcomes and exposures (Table S6). Additionally, to further strengthen the causal associations between exposure and outcomes, we conducted reverse MR enhancing the credibility of our results (Table S7). Sensitivity analyses were conducted for all significant results, including heterogeneity analyses (Table S8), horizontal pleiotropy analyses (Table S9), funnel plots (Figure S7), and leave‐one‐out analyses (Figure S8).

#### MVMR analyses

Before performing the MVMR analyses, we conducted an initial screening of risk factors linked to liver enzymes using bidirectional UVMR. The adjustment factors for each liver enzyme used in model 1 MVMR analyses were confirmed as follows: HT and TC for AST; SI, DPW, HT, and TC for ALP; BMI, T2DM, SI, and TC for ALT; and BMI, T2DM, SI, HT, DPW, and TC for GGT (Table S10).

**FIGURE 6:**
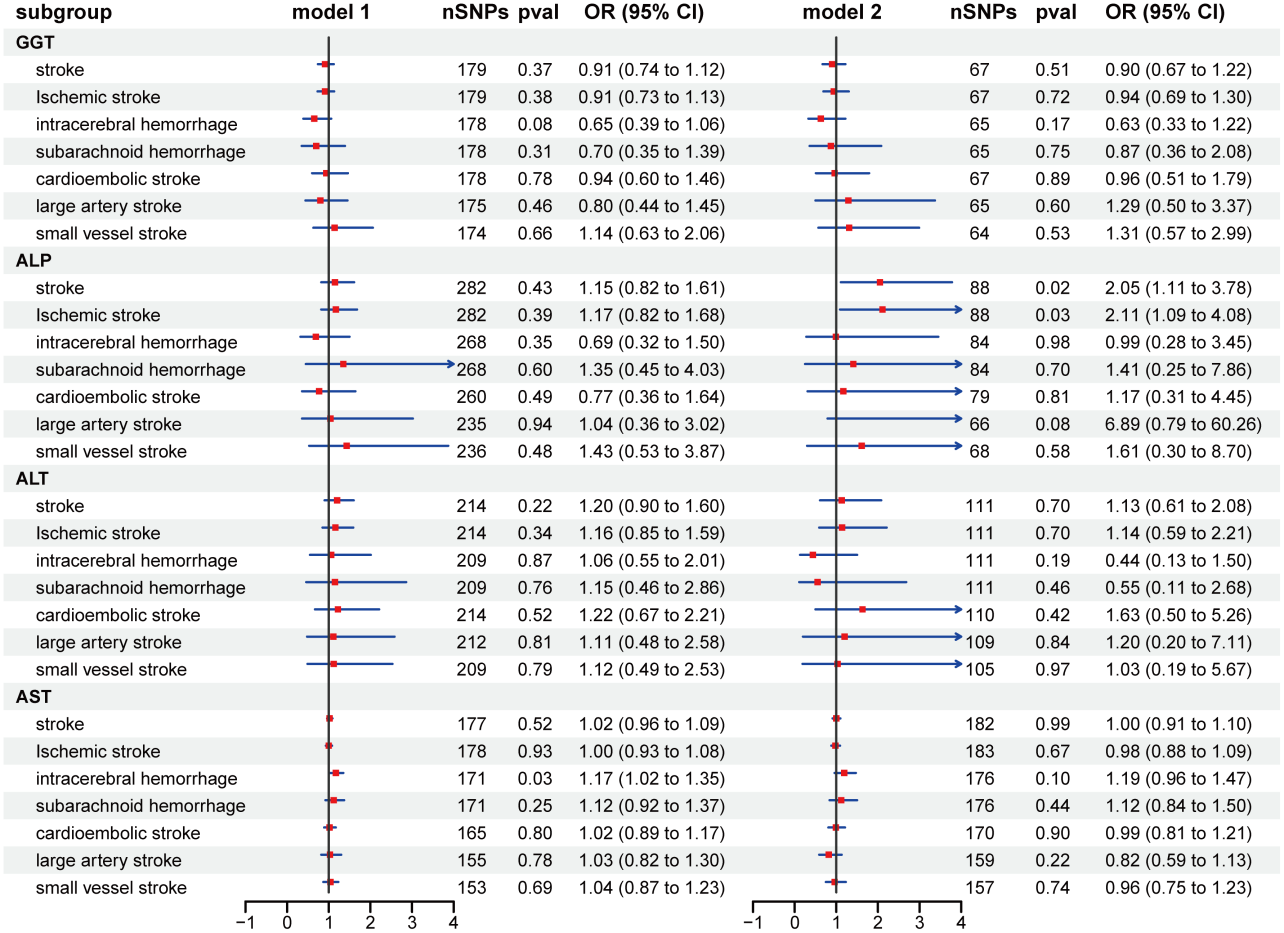
Multivariable Mendelian randomization analyses of the association between liver enzymes and the risk of stroke and its subtypes. Squares represent the estimate of odds ratio, and the horizontal lines represent the 95% confidence interval. ALP, alkaline phosphatase; ALT, alanine aminotransferase; AST, aspartate aminotransferase; CI, confidence interval; GGT, γ‐glutamyl transferase; MVMR, multivariable Mendelian randomization; OR, odds ratio; SNP, single‐nucleotide polymorphism.

The results of the MVMR analyses were shown in Figure 6 and detailed in Table S11. Based on model 1, the MVMR analyses unveiled a notable causal association between AST and ICH (nSNPs = 171, OR: 1.17, 95% CI: 1.02–1.35, *p* = 0.025). Findings from model 2 MVMR analyses, which included additional adjustments for the other three liver enzymes, demonstrated significant causal links between ALP with stroke (nSNPs = 88, OR: 2.05, 95% CI: 1.11–3.78, *p* = 0.021) and IS (nSNPs = 88, OR: 2.11, 95% CI: 1.09–4.08, *p* = 0.027).

#### Gene set and tissue enrichment analyses

To explore potential sex disparities in genetic variations of liver enzymes, we conducted gene annotation (Table S12), gene set enrichment analyses, and tissue enrichment analyses for significant SNPs across sexes. The gene set enrichment analyses unveiled variances in lipid metabolism, immune processes, and protein regulation (Figure S9). Similarly, tissue enrichment results also highlighted sex disparities. For instance, SNPs linked to ALP were predominantly enriched in the spleen, blood, and skin among females, whereas males exhibited supplementary enrichment in the liver and kidneys (Figure S10).

## DISCUSSION

This study investigated an association between liver enzymes and the risk of stroke based on observational and genetic data. The observational analyses revealed a significant association between elevated liver enzyme levels and an increased risk of stroke, with notable differences observed across sexes and stroke subtypes. Moreover, the MR analyses provided strong evidence for a causal relationship, reinforcing the validity of the observed associations. These findings underscore the potential of liver enzymes as biomarkers for stroke risk stratification, highlighting their value in guiding early intervention strategies.

A previous meta‐analysis, comprising 5707 cases and 926,497 participants, has identified a notable association between elevated GGT levels and heightened stroke risk [34]. In our expanded meta‐analyses, comprising 35,484 cases and 2,330,353 controls, this association was further corroborated. As a regulator of glutathione metabolism, GGT plays a crucial role in maintaining antioxidant homeostasis by recycling extracellular glutathione, which is essential for metabolic processes [5]. Elevated serum GGT levels are indicative of enhanced inflammatory states and increased oxidative stress [35]. Consequently, GGT may accelerate atherosclerosis progression via oxidative and inflammatory mechanisms [36]. Additionally, several studies have reported an association between GGT and atrial fibrillation, attributed to oxidative stress, chronic low‐grade inflammation, and metabolic syndrome [36, 37]. This suggests a potential overlap between elevated GGT levels and the underlying mechanisms of stroke occurrence, which could help elucidate how elevated GGT contributes to an increased stroke risk. In our stratified analyses, sex disparities were observed in the correlation between GGT levels and HS risk, particularly indicating a correlation with ICH risk in males rather than females. Several factors may contribute to this observation. First, ICH is more prevalent in males [38]. Second, a combination of genetic and environmental factors substantially influences the biological effects of liver enzyme levels, encompassing metabolic risk factors, inflammatory markers, alcohol consumption, smoking habits, and coffee intake [39]. In our two‐sample bidirectional UVMR analyses, we found an association between GGT and CES risk; however, this association was attenuated in MVMR analyses. The discrepancy with previous findings [36] may be attributed to our more stringent selection of covariates. The risk associated with GGT levels predominantly affected CES and was bolstered by mechanisms through which GGT fostered atherosclerosis. Overall, the influence of GGT on stroke risk was noticeable, albeit genetically, and its risk was mainly linked to CES.

ALP is a widely expressed enzyme, yet it has received relatively less attention. Elevated ALP levels may indicate increased bone metabolic activity, which can accelerate vascular calcification, potentially promoting atherosclerosis progression, vascular aging, and even vascular rupture [40]. Increased bone metabolism activity and the promotion of vascular homeostasis are mechanisms that may elucidate why elevated ALP levels could pose a risk factor for stroke, particularly IS [41]. Previous studies indicate that serum ALP levels typically elevate with age, especially among postmenopausal women [42, 43]. Therefore, the sex differences observed in our findings might be linked to several factors contributing to heightened ALP levels, including age, female, bone disorders, chronic kidney disease (CKD), and inflammation [43]. Interestingly, females exhibited a higher incidence of SAH but a lower incidence of IS. The elevated prevalence of SAH in females may have contributed to elucidating the result [44].

The results from the MVMR analysis, consistent with our meta‐analysis findings, emphasize the role of ALP as a risk factor for stroke. The sex specificity of stroke etiology and the multiple tissue sources of ALP isoenzymes may be the primary determinant of sex‐specific differences. Gene set enrichment analyses and tissue enrichment analyses of SNPs significantly associated with ALP confirmed the presence of genetic effects contributing to sex differences in ALP activity. Furthermore, a newly developed method for quantifying ALP activity and isoenzyme composition in blood provides a robust tool for future ALP research, enabling rapid and accurate identification of various health conditions in patients [45].

ALT and AST are primary serum aminotransferases and sensitive indicators of liver damage. Consistent with prior studies [46], our meta‐analyses did not reveal a significant association between these enzymes and the overall risk of stroke. The discrepancies observed in the stratified analysis of ALT may be attributed to our sample size, which was more than twice that of previous studies, resulting in more robust conclusions. The Atherosclerosis Risk in Communities (ARIC) study, involving 12,588 participants aged 45–64 years, reported that elevated AST levels were associated with an increased risk of HS, but not IS, while elevated ALT levels were not linked to stroke risk [47]. Compared with the results, we additionally identified new evidence linking ALT to an increased risk of ICH in males. This association may be attributed to several risk factors that predispose individuals to ICH, including age, male, Asian ancestry, CKD, cerebral amyloid angiopathy, and cerebral microbleeds [48]. Both ALT and AST catalyze the transfer of amino groups from amino acids to α‐ketoglutarate, and due to the importance of their substrates and products in numerous cellular processes, they play vital physiological roles beyond amino acid metabolism [7]. Notably, they are also essential for maintaining energy homeostasis [7]. Elevated serum ALT has been identified as an independent marker of systemic inflammation and increased oxidative stress, which may contribute to vascular fragility and the occurrence of hemorrhagic events [35]. Serum measurements do not differentiate between these isoforms, which may be a key factor contributing to the inconsistencies observed across studies. In our UVMR analyses, we identified an association between ALT levels and the risks of stroke and IS, aligning with previous MR results [17]. However, no association was discovered in MVMR analyses, echoing the outcomes of our meta‐analyses, indicating a comparatively weaker correlation between ALT and stroke risk.

Our study has several highlights. Firstly, in the meta‐analyses, we analyzed data from over 2 million individuals and extended earlier findings through comprehensive stratification based on sexes and stroke subtypes [46]. Secondly, in our MR analyses, we employed the largest publicly available GWAS dataset to minimize the likelihood of insufficient statistical power, and conducted both UVMR and MVMR analyses to explore the direct genetic causal relationships between liver enzymes and stroke risk. Finally, and most importantly, this study offers notable public health and clinical guidance. Previous studies have mostly focused on exploring the potential biomarker of ALP as prognostic tools, noting an association between elevated ALP levels and increased mortality in stroke patients, or identifying a correlation between ALP and stroke incidence among high‐risk individuals [49, 50]. However, our study underscores the prospective role of ALP as a predictive biomarker for stroke risk in the general population, which could assist clinicians in identifying and intervening in high‐risk groups.

However, several limitations should be noted. (i) The included studies did not adequately consider potential confounding factors that could affect liver enzyme levels, such as sleep duration and quality, medication use, and diet, which may have introduced bias into the results. (ii) Subgroup analyses in the meta‐analyses are hindered by limited statistical power due to the few studies in each stratum, necessitating further research to confirm result robustness. (iii) Lack of sex‐specific causal evidence is due to limitations in stroke GWAS data stratified by sex. (iv) MR analyses are restricted to European populations, potentially limiting the generalizability of the study findings. Furthermore, our exposure discovery cohort is derived from the UKB, which restricts the inclusion of data from European ancestral populations in other countries. However, these datasets represent the largest sample of European ancestry available in liver enzymes GWAS analyses. We anticipate that the substantial sample size here could partially compensate for the lack of population diversity. Future GWAS meta‐analyses on liver enzymes and sex‐stratified stroke GWAS data analyses in populations from additional countries and regions will contribute to further validating our findings.

## CONCLUSIONS

The study employed a comprehensive research design integrating meta‐analyses and MR analyses to enhance causal inference regarding liver enzymes and stroke risk, considering population‐based and genetic perspectives. Through rigorous updating, validation, and exploration, our study confirmed the link between liver enzymes and stroke risk, emphasizing the imperative for heightened attention to ALP. In the future, further elucidation of the mechanisms linking liver enzyme levels to stroke risk may benefit the identification and intervention of high‐risk populations.

## AUTHOR CONTRIBUTIONS

**Chun Li:** Writing – original draft; conceptualization; formal analysis; investigation. **Long Gu:** Writing – original draft; conceptualization; software; methodology. **Fu‐Yi Shi:** Data curation; validation. **Shi‐Ying Xiong:** Formal analysis; validation. **Gui‐Sheng Wu:** Formal analysis; funding acquisition. **Jian‐Hua Peng:** Validation. **Ruo‐Lan Wang:** Software. **Yuan Yuan:** Software. **Yong Jiang:** Writing – review and editing; project administration; supervision. **Chen Huang:** Writing – review and editing; resources; supervision. **Huai‐Rong Luo:** Writing – review and editing; supervision; funding acquisition.

## FUNDING INFORMATION

This work was supported by grants from the National Natural Science Foundation of China (82171555), Central Nervous System Drug Key Laboratory of Sichuan Province (230003‐01SZ), Development and Application of Human Major Disease Monkey Model Key Laboratory of Sichuan Province (2023KF002), and Luzhou Science and Technology Program (2022SWMU4).

## CONFLICT OF INTEREST STATEMENT

All authors declare no competing interests.

## Supporting information


Supporting Information File 1.

Supporting Information File 2.

Supporting Information File 3.

Supporting Information File 4.

Supporting Information File 5.

## Data Availability

The meta‐analyses data in this study were derived from published literature, with detailed references provided in Supporting Information File 3. Datasets for GWAS summary statistics on liver enzymes are publicly accessible via the NHGRI‐EBI GWAS Catalog (https://www.ebi.ac.uk/gwas/), with accession codes GCST90013405‐GCST90013407, and GSCT90025980. GWAS summary statistics for stroke, IS and its subtypes, can be found in the NHGRI‐EBI GWAS Catalog under accession codes GCST90104539‐GCST90104543. GWAS summary statistics for SAH and ICH are accessible through the FinnGen R10 release (https://www.finngen.fi/en/access_results), with phenocodes I9_SAH and I9_INTRACRA, respectively. Other GWAS data sources are detailed in Table S2.

## References

[1] Ding Q , Liu S , Yao Y , Liu H , Cai T , Han L . Global, regional, and national burden of ischemic stroke, 1990–2019. Neurology. 2022;98(3):e279‐e290. doi:10.1212/WNL.0000000000013115 34911748

[2] Collaborators GBDS . Global, regional, and national burden of stroke and its risk factors, 1990–2019: a systematic analysis for the global burden of disease study 2019. Lancet Neurol. 2021;20(10):795‐820. doi:10.1016/S1474-4422(21)00252-0 34487721 PMC8443449

[3] Pandian JD , Gall SL , Kate MP , et al. Prevention of stroke: a global perspective. Lancet. 2018;392(10154):1269‐1278. doi:10.1016/S0140-6736(18)31269-8 30319114

[4] Tamber SS , Bansal P , Sharma S , Singh RB , Sharma R . Biomarkers of liver diseases. Mol Biol Rep. 2023;50(9):7815‐7823. doi:10.1007/s11033-023-08666-0 37482588

[5] Mitrić A , Castellano I . Targeting gamma‐glutamyl transpeptidase: a pleiotropic enzyme involved in glutathione metabolism and in the control of redox homeostasis. Free Radic Biol Med. 2023;208:672‐683. doi:10.1016/j.freeradbiomed.2023.09.020 37739139

[6] Zaher DM , El‐Gamal MI , Omar HA , et al. Recent advances with alkaline phosphatase isoenzymes and their inhibitors. Arch Pharm (Weinheim). 2020;353(5):e2000011. doi:10.1002/ardp.202000011 32128876

[7] McGill MR . The past and present of serum aminotransferases and the future of liver injury biomarkers. EXCLI J. 2016;15:817‐828. doi:10.17179/excli2016-800 28337112 PMC5318690

[8] Arafa A , Kokubo Y , Kashima R , Matsumoto C , Koga M . Liver enzymes and the risk of stroke among the general Japanese population: a prospective cohort study. Cerebrovasc Dis. 2023;53:252‐260. doi:10.1159/000533654 37591215

[9] Yang YJ , Jung MH , Jeong SH , Hong YP , Kim YI , An SJ . The association between nonalcoholic fatty liver disease and stroke: results from the Korean Genome and Epidemiology Study (KoGES). Int J Environ Res Public Health. 2020;17(24):9568. doi:10.3390/ijerph17249568 33371282 PMC7765788

[10] Sekula P , Del Greco MF , Pattaro C , Kottgen A . Mendelian randomization as an approach to assess causality using observational data. J Am Soc Nephrol. 2016;27(11):3253‐3265. doi:10.1681/ASN.2016010098 27486138 PMC5084898

[11] Page MJ , McKenzie JE , Bossuyt PM , et al. The PRISMA 2020 statement: an updated guideline for reporting systematic reviews. Rev Esp Cardiol (Engl ed). 2021;74(9):790. doi:10.1016/j.rec.2021.07.010 34446261

[12] Skrivankova VW , Richmond RC , Woolf BAR , et al. Strengthening the reporting of observational studies in epidemiology using mendelian randomisation (STROBE‐MR): explanation and elaboration. BMJ. 2021;375:n2233. doi:10.1136/bmj.n2233 34702754 PMC8546498

[13] Wells, GA , Shea, B , O'Connell, D , et al. The Newcastle‐Ottawa Scale (NOS) for assessing the quality of nonrandomised studies in meta‐analyses. Ottawa Hospital Research Institute. 2000. www.ohri.ca/programs/clinical_epidemiology/oxford.asp

[14] Brunström M , Carlberg B . Effect of antihypertensive treatment at different blood pressure levels in patients with diabetes mellitus: systematic review and meta‐analyses. BMJ. 2016;352:i717. doi:10.1136/bmj.i717 26920333 PMC4770818

[15] Borenstein M . Common Mistakes in Meta‐Analysis and How to Avoid Them. Biostat, Inc; 2019.

[16] Cai X , Zhang Y , Li M , et al. Association between prediabetes and risk of all cause mortality and cardiovascular disease: updated meta‐analysis. BMJ. 2020;370:m2297. doi:10.1136/bmj.m2297 32669282 PMC7362233

[17] Pazoki R , Vujkovic M , Elliott J , et al. Genetic analysis in European ancestry individuals identifies 517 loci associated with liver enzymes. Nat Commun. 2021;12(1):2579. doi:10.1038/s41467-021-22338-2 33972514 PMC8110798

[18] Barton AR , Sherman MA , Mukamel RE , Loh P‐R . Whole‐exome imputation within UK biobank powers rare coding variant association and fine‐mapping analyses. Nat Genet. 2021;53(8):1260‐1269. doi:10.1038/s41588-021-00892-1 34226706 PMC8349845

[19] Mishra A , Malik R , Hachiya T , et al. Stroke genetics informs drug discovery and risk prediction across ancestries. Nature. 2022;611(7934):115‐123. doi:10.1038/s41586-022-05165-3 36180795 PMC9524349

[20] Kurki MI , Karjalainen J , Palta P , et al. FinnGen provides genetic insights from a well‐phenotyped isolated population. Nature. 2023;613(7944):508‐518. doi:10.1038/s41586-022-05473-8 36653562 PMC9849126

[21] Pulit SL , Stoneman C , Morris AP , et al. Meta‐analysis of genome‐wide association studies for body fat distribution in 694 649 individuals of European ancestry. Hum Mol Genet. 2019;28(1):166‐174. doi:10.1093/hmg/ddy327 30239722 PMC6298238

[22] Zhu Z , Wang X , Li X , et al. Genetic overlap of chronic obstructive pulmonary disease and cardiovascular disease‐related traits: a large‐scale genome‐wide cross‐trait analysis. Respir Res. 2019;20(1):64. doi:10.1186/s12931-019-1036-8 30940143 PMC6444755

[23] Xue A , Wu Y , Zhu Z , et al. Genome‐wide association analyses identify 143 risk variants and putative regulatory mechanisms for type 2 diabetes. Nat Commun. 2018;9:2941. doi:10.1038/s41467-018-04951-w 30054458 PMC6063971

[24] Saunders GRB , Wang X , Chen F , et al. Genetic diversity fuels gene discovery for tobacco and alcohol use. Nature. 2022;612(7941):720‐724. doi:10.1038/s41586-022-05477-4 36477530 PMC9771818

[25] Graham SE , Clarke SL , Wu KH , et al. The power of genetic diversity in genome‐wide association studies of lipids. Nature. 2021;600(7890):675‐679. doi:10.1038/s41586-021-04064-3 34887591 PMC8730582

[26] Myers TA , Chanock SJ , Machiela MJ . LDlinkR: an R package for rapidly calculating linkage disequilibrium statistics in diverse populations. Front Genet. 2020;11:157. doi:10.3389/fgene.2020.00157 32180801 PMC7059597

[27] Yates AD , Allen J , Amode RM , et al. Ensembl genomes 2022: an expanding genome resource for non‐vertebrates. Nucleic Acids Res. 2022;50(D1):D996‐D1003. doi:10.1093/nar/gkab1007 34791415 PMC8728113

[28] Verbanck M , Chen C‐Y , Neale B , Do R . Detection of widespread horizontal pleiotropy in causal relationships inferred from Mendelian randomization between complex traits and diseases. Nat Genet. 2018;50(5):693‐698. doi:10.1038/s41588-018-0099-7 29686387 PMC6083837

[29] Burgess S , Davies NM , Thompson SG . Bias due to participant overlap in two‐sample mendelian randomization. Genet Epidemiol. 2016;40(7):597‐608. doi:10.1002/gepi.21998 27625185 PMC5082560

[30] Sanderson E . Multivariable Mendelian randomization and mediation. Cold Spring Harb Perspect Med. 2021;11(2):a038984. doi:10.1101/cshperspect.a038984 32341063 PMC7849347

[31] Hemani G , Zheng J , Elsworth B , et al. The MR‐base platform supports systematic causal inference across the human phenome. elife. 2018;7:e34408. doi:10.7554/eLife.34408 29846171 PMC5976434

[32] Watanabe K , Taskesen E , van Bochoven A , Posthuma D . Functional mapping and annotation of genetic associations with FUMA. Nat Commun. 2017;8(1):1826. doi:10.1038/s41467-017-01261-5 29184056 PMC5705698

[33] Wu T , Hu E , Xu S , et al. clusterProfiler 4.0: a universal enrichment tool for interpreting omics data. Innovation (Camb). 2021;2(3):100141. doi:10.1016/j.xinn.2021.100141 34557778 PMC8454663

[34] Zhang X‐W , Li M , Hou W‐S , Li K , Zhou J‐R , Tang Z‐Y . Association between gamma‐glutamyltransferase level and risk of stroke: a systematic review and meta‐analysis of prospective studies. J Stroke Cerebrovasc Dis. 2015;24(12):2816‐2823. doi:10.1016/j.jstrokecerebrovasdis.2015.08.015 26372100

[35] Yamada J , Tomiyama H , Yambe M , et al. Elevated serum levels of alanine aminotransferase and gamma glutamyltransferase are markers of inflammation and oxidative stress independent of the metabolic syndrome. Atherosclerosis. 2006;189(1):198‐205. 16405892 10.1016/j.atherosclerosis.2005.11.036

[36] Lee Y , Seo JH . Potential causal association between elevated gamma‐glutamyl transferase level and stroke: a two‐sample Mendelian randomization study. Biomol Ther. 2023;13(11):1592. doi:10.3390/biom13111592 PMC10669367 38002274

[37] Roychoudhury R , Ma S , Qian C . Stroke prevention and intracranial hemorrhage risk in atrial fibrillation management: a mini review. Brain Circ. 2023;9(3):148‐153. doi:10.4103/bc.bc_22_23 38020950 PMC10679623

[38] Poon MT , Bell SM , Al‐Shahi Salman R . Epidemiology of intracerebral haemorrhage. Front Neurol Neurosci. 2015;37:1‐12. doi:10.1159/000437109 26588164

[39] van Beek JH , de Moor MH , de Geus EJ , et al. The genetic architecture of liver enzyme levels: GGT, ALT and AST. Behav Genet. 2013;43(4):329‐339. doi:10.1007/s10519-013-9593-y 23580007 PMC3918238

[40] Liu K , Yu Y , Yuan Y , et al. Elevated levels of serum alkaline phosphatase are associated with increased risk of cardiovascular disease: a prospective cohort study. J Atheroscler Thromb. 2023;30(7):795‐819. doi:10.5551/jat.63646 36261365 PMC10322736

[41] Shimizu Y , Imano H , Ohira T , et al. Alkaline phosphatase and risk of stroke among Japanese: the circulatory risk in communities study (CIRCS). J Stroke Cerebrovasc Dis. 2013;22(7):1046‐1055. doi:10.1016/j.jstrokecerebrovasdis.2012.06.009 22841505

[42] Pratt DS , Kaplan MM . Evaluation of abnormal liver‐enzyme results in asymptomatic patients. N Engl J Med. 2000;342(17):1266‐1271. doi:10.1056/NEJM200004273421707 10781624

[43] Kwo PY , Cohen SM , Lim JK . ACG clinical guideline: evaluation of abnormal liver chemistries. Am J Gastroenterol. 2017;112(1):18‐35. doi:10.1038/ajg.2016.517 27995906

[44] Kalasapudi L , Williamson S , Shipper AG , et al. Scoping review of racial, ethnic, and sex disparities in the diagnosis and management of hemorrhagic stroke. Neurology. 2023;101(3):e267‐e276. doi:10.1212/WNL.0000000000207406 37202159 PMC10382273

[45] Jiang Y , Li X , Walt DR . Single‐molecule analysis determines isozymes of human alkaline phosphatase in serum. Angew Chem Int Ed Engl. 2020;59(41):18010‐18015. doi:10.1002/anie.202007477 32613710

[46] Kunutsor SK , Apekey TA , Khan H . Liver enzymes and risk of cardiovascular disease in the general population: a meta‐analysis of prospective cohort studies. Atherosclerosis. 2014;236(1):7‐17. doi:10.1016/j.atherosclerosis.2014.06.006 24998934

[47] Ruban A , Daya N , Schneider ALC , et al. Liver enzymes and risk of stroke: the Atherosclerosis Risk in Communities (ARIC) study. J Stroke. 2020;22(3):357‐368. doi:10.5853/jos.2020.00290 33053951 PMC7568972

[48] An SJ , Kim TJ , Yoon BW . Epidemiology, risk factors, and clinical features of intracerebral hemorrhage: an update. J Stroke. 2017;19(1):3‐10. doi:10.5853/jos.2016.00864 28178408 PMC5307940

[49] Duan H , Geng X , Ding Y . Hepatic responses following acute ischemic stroke: a clinical research update. Brain Circ. 2023;9(2):57‐60. doi:10.4103/bc.bc_31_23 37576577 PMC10419733

[50] Zong L , Wang X , Li Z , et al. Alkaline phosphatase and outcomes in patients with preserved renal function: results from China National Stroke Registry. Stroke. 2018;49(5):1176‐1182. doi:10.1161/STROKEAHA.118.020237 29669879

