# Supplementary material for: Serum liver enzymes and risk of stroke: Systematic review with meta‐analyses and Mendelian randomization studies
Source: Eur J Neurol. 2024 Oct 10;31(12):e16506. doi: 10.1111/ene.16506 (PMC11555028; doi:10.1111/ene.16506)
Supplement: Supplementary file 2 — Supporting Information File 2. [file ENE-31-e16506-s002.docx]

**STROBE-MR checklist of recommended items to address in reports of Mendelian randomization studies**^1^ ^2^

| **Item No.** | **Section** | **Checklist item** | **Page No.** | **Relevant text from manuscript** |
| --- | --- | --- | --- | --- |
| 1 | **TITLE and ABSTRACT** | Indicate Mendelian randomization (MR) as the study’s design in the title and/or the abstract if that is a main purpose of the study | 1 | Serum Liver Enzymes and Risk of Stroke: Systematic Review with Meta-analyses and Mendelian Randomization Studies |
|  | **INTRODUCTION** |  |  |  |
| 2 | **Background** | Explain the scientific background and rationale for the reported study. What is the exposure? Is a potential causal relationship between exposure and outcome plausible? Justify why MR is a helpful method to address the study question | 3-4 | Stroke is a prominent contributor to early mortality and enduring incapacitation on a global scale1. The fatalities, cognitive decline, and impairments induced by stroke worldwide obviously diminish life quality and burden economies and societies, rendering it a substantial public health concern. The increasing burden and costs associated with stroke management underscore the urgent need for effective stroke prevention strategies. It is noteworthy that over 90% of stroke cases arise from potentially modifiable risk factors, with more than 75% of this burden being potentially reducible through the management of metabolic and behavioral risk factors.  Multiple studies have found associations between liver enzyme levels and the risk of stroke; nonetheless, the results remain controversial and inconclusive.  Serum liver enzymes, encompassing γ-glutamyl transferase (GGT), alkaline phosphatase (ALP), alanine aminotransferase (ALT), and aspartate aminotransferase (AST), are commonly utilized as clinical indicators for evaluating liver function. However, these enzymes are not exclusively indicative of liver pathology.  In observational studies, inevitable confounding factors affect the causal inference of liver enzyme levels in stroke risk. Consequently, genetic analyses independent of external factors and disease progression might mitigate confounding effects and reverse causality. Mendelian randomization (MR), an epidemiological method, aids in establishing causal relationships in exposure-outcome associations by employing genetic variation as instrumental variables for exposure. |
| 3 | **Objectives** | State specific objectives clearly, including pre-specified causal hypotheses (if any). State that MR is a method that, under specific assumptions, intends to estimate causal effects | 4 | Here, we conducted meta-analyses to summarize existing evidence from traditional observational studies and performed two-sample bidirectional univariable Mendelian randomization (UVMR) and multivariable Mendelian randomization (MVMR) analyses to investigate the association between four liver enzyme levels and stroke risk. Our aim was to provide a comprehensive evaluation of these associations, thereby contributing to the foundation for stroke prevention strategies. |
|  | **METHODS** |  |  |  |
| 4 | **Study design and data sources** | Present key elements of the study design early in the article. Consider including a table listing sources of data for all phases of the study. For each data source contributing to the analysis, describe the following: |  |  |
|  | a) | Setting: Describe the study design and the underlying population, if possible. Describe the setting, locations, and relevant dates, including periods of recruitment, exposure, follow-up, and data collection, when available. | 6 | Summary data for serum levels of three liver enzymes (ALT, ALP, GGT) were collected from a genome-wide association studies (GWASs) including 437,438 individuals of European ancestry. Serum AST data was obtained from the GWAS data including 436,275 individuals of European ancestry from the UK Biobank (UKB). GWAS summary data related to stroke were acquired from the GIGASTROKE consortium, including 73,652 stroke cases and 1,234,808 controls of European ancestry. The GIGASTROKE study encompasses 62,100 cases of ischemic stroke (IS), systematically identified according to the Trial of ORG 10172 in Acute Stroke Treatment (TOAST) criteria, and three distinct IS subtypes were classified: large vessel atherosclerosis stroke (LVS, n = 6,399), small vessel occlusion stroke (SVS, n = 6,811), and cardioembolic stroke (CES, n = 10,804). GWAS data for subarachnoid hemorrhage (SAH) and intracerebral hemorrhage (ICH) were derived from FinnGen, with SAH comprising 3,532 cases and 371,753 controls, and ICH comprising 7,040 cases and 374,631 controls. |
|  | b) | Participants: Give the eligibility criteria, and the sources and methods of selection of participants. Report the sample size, and whether any power or sample size calculations were carried out prior to the main analysis | 6, 17 | The sources and relevant information of the GWAS data are detailed in Table S2. |
|  | c) | Describe measurement, quality control and selection of genetic variants | 6-7 | Single-nucleotide polymorphisms (SNPs) closely associated with serum enzyme activity of individual liver enzymes were identified using a significance threshold of 5e−8. This threshold was chosen to ensure strong instrument relevance while minimizing the risk of false positives, a standard practice in large-scale GWAS-based MR analyses. Linkage disequilibrium (LD) among SNPs was alleviated (r^2^ < 0.001, kb = 1,000) to ensure the independence of instrumental variables. This additional step was critical to ensure that the selected SNPs were not associated with potential confounders, thus addressing the exchangeability assumption in MR analysis. |
|  | d) | For each exposure, outcome, and other relevant variables, describe methods of assessment and diagnostic criteria for diseases | NA |  |
|  | e) | Provide details of ethics committee approval and participant informed consent, if relevant | NA |  |
| 5 | **Assumptions** | Explicitly state the three core IV assumptions for the main analysis (relevance, independence and exclusion restriction) as well assumptions for any additional or sensitivity analysis | 6-7 | We rigorously uphold the three key assumptions: relevance, exchangeability, and exclusion restriction principle, during the execution of our MR analysis. |
| 6 | **Statistical methods: main analysis** | Describe statistical methods and statistics used |  |  |
|  | a) | Describe how quantitative variables were handled in the analyses (i.e., scale, units, model) | NA |  |
|  | b) | Describe how genetic variants were handled in the analyses and, if applicable, how their weights were selected | NA |  |
|  | c) | Describe the MR estimator (e.g. two-stage least squares, Wald ratio) and related statistics. Detail the included covariates and, in case of two-sample MR, whether the same covariate set was used for adjustment in the two samples | 7 | Additionally, GWAS summary data for total cholesterol levels (TC), hypertension (HT), smoking initiation (SI), drinks per week (DPW), type 2 diabetes mellitus (T2DM), and BMI were obtained for adjusting confounding factors. |
|  | d) | Explain how missing data were addressed | NA |  |
|  | e) | If applicable, indicate how multiple testing was addressed | 8 | A *P*-threshold of 0.05/3 (number of stroke subtypes or IS subtypes) was defined as statistical significance. |
| 7 | **Assessment of assumptions** | Describe any methods or prior knowledge used to assess the assumptions or justify their validity | 8 | Additionally, we performed pleiotropy tests, constructed funnel plots, and conducted leave-one-out analyses to ensure the fulfillment of the key assumptions of MR. |
| 8 | **Sensitivity analyses and additional analyses** | Describe any sensitivity analyses or additional analyses performed (e.g. comparison of effect estimates from different approaches, independent replication, bias analytic techniques, validation of instruments, simulations) | 7-8 | To address potential pleiotropy, we employed several sensitivity analyses, including tests for heterogeneity, MR-Egger regression, and the weighted median method. These methods are specifically designed to detect and adjust for pleiotropy, ensuring that any observed associations are not driven by pleiotropic effects of the SNPs. MR-Egger, for example, can test for directional pleiotropy, while the weighted median method can provide reliable estimates even when up to 50% of the SNPs are invalid. |
| 9 | **Software and pre-registration** |  |  |  |
|  | a) | Name statistical software and package(s), including version and settings used | 7 | Analyses were performed using R packages TwoSampleMR (version 0.5.8) 27, MR-PRESSO (version 1.0), and LDlinkR (version 1.3.0) 22 in R version 4.2.3. A *P*-threshold of 0.05/3 (number of stroke subtypes or IS subtypes) was defined as statistical significance. |
|  | b) | State whether the study protocol and details were pre-registered (as well as when and where) | NA |  |
|  | **RESULTS** |  |  |  |
| 10 | **Descriptive data** |  |  |  |
|  | a) | Report the numbers of individuals at each stage of included studies and reasons for exclusion. Consider use of a flow diagram | NA |  |
|  | b) | Report summary statistics for phenotypic exposure(s), outcome(s), and other relevant variables (e.g. means, SDs, proportions) |  | Table S2. |
|  | c) | If the data sources include meta-analyses of previous studies, provide the assessments of heterogeneity across these studies | NA |  |
|  | d) | For two-sample MR:  i.  Provide justification of the similarity of the genetic variant-exposure associations between the exposure and outcome samples  ii.  Provide information on the number of individuals who overlap between the exposure and outcome studies |  | \| i. We utilized distinct data sources for exposures and outcomes, both derived from individuals of European ancestry. \| \| --- \| \| ii. As summary-level statistics were employed, it was not feasible to identify individuals who overlapped between the exposure and outcome datasets. \| |
| 11 | **Main results** |  |  |  |
|  | a) | Report the associations between genetic variant and exposure, and between genetic variant and outcome, preferably on an interpretable scale |  | Table S3. |
|  | b) | Report MR estimates of the relationship between exposure and outcome, and the measures of uncertainty from the MR analysis, on an interpretable scale, such as odds ratio or relative risk per SD difference | 11 | We observed that AST activity levels exhibited a statistical significant causal effect on SAH (Figure 5A, nSNPs = 189, OR: 1.35, 95% CI: 1.12-1.64, *p* = 0.002) and ICH (Figure 5B, nSNPs = 189, OR: 1.22, 95% CI: 1.06-1.40, *p* = 0.005). ALT showed nominal significant associations with stroke (Figure 5C, nSNPs = 130, OR: 1.73, 95% CI: 1.10-2.70, *p* = 0.017) and IS (Figure 5D, nSNPs = 131, OR: 1.71, 95% CI: 1.03-2.84, *p* = 0.038). Additionally, the results revealed a nominal significant causal relationship between GGT and CES risk (Figure 5E, nSNPs = 227, OR: 1.50, 95% CI: 1.03-2.18, *p* = 0.033). No nominal significant causal associations were observed between other outcomes and exposures (Table S6). |
|  | c) | If relevant, consider translating estimates of relative risk into absolute risk for a meaningful time period | NA |  |
|  | d) | Consider plots to visualize results (e.g. forest plot, scatterplot of associations between genetic variants and outcome versus between genetic variants and exposure) |  | Figure 5. |
| 12 | **Assessment of assumptions** |  |  |  |
|  | a) | Report the assessment of the validity of the assumptions | 12 | Sensitivity analyses were conducted for all significant results, including heterogeneity analyses (Table S8), horizontal pleiotropy analyses (Table S9), funnel plots (Figure S7), and leave-one-out analyses (Figure S8). |
|  | b) | Report any additional statistics (e.g., assessments of heterogeneity across genetic variants, such as *I^2^*, Q statistic or E-value) | 11 | We estimated the phenotypic variance explained by R^2^ and calculated the *F*-statistics to assess the strength of instrumental variables (Table S3). |
| 13 | **Sensitivity analyses and additional analyses** |  |  |  |
|  | a) | Report any sensitivity analyses to assess the robustness of the main results to violations of the assumptions | 11 | A causal inference was inferred when an effect estimate achieved statistical significance in the IVW analysis and maintained directional consistency across the other analytical approaches. |
|  | b) | Report results from other sensitivity analyses or additional analyses | NA |  |
|  | c) | Report any assessment of direction of causal relationship (e.g., bidirectional MR) | 12 | Additionally, to further strengthen the causal associations between exposure and outcomes, we conducted reverse MR enhancing the credibility of our results (Table S7). |
|  | d) | When relevant, report and compare with estimates from non-MR analyses | NA |  |
|  | e) | Consider additional plots to visualize results (e.g., leave-one-out analyses) |  | Figures S7-8 |
|  | **DISCUSSION** |  |  |  |
| 14 | **Key results** | Summarize key results with reference to study objectives | 13 | This study investigated an association between liver enzymes and the risk of stroke based on observational and genetic data. The observational analyses revealed a significant association between elevated liver enzyme levels and an increased risk of stroke, with notable differences observed across sexes and stroke subtypes. Moreover, the MR analyses provided strong evidence for a causal relationship, reinforcing the validity of the observed associations. These findings underscore the potential of liver enzymes as biomarkers for stroke risk stratification, highlighting their value in guiding early intervention strategies. |
| 15 | **Limitations** | Discuss limitations of the study, taking into account the validity of the IV assumptions, other sources of potential bias, and imprecision. Discuss both direction and magnitude of any potential bias and any efforts to address them | 16-17 | Furthermore, our exposure discovery cohort is derived from the UKB, which restricts the inclusion of data from European ancestral populations in other countries. However, these datasets represent the largest sample of European ancestry available in liver enzymes GWAS analyses. We anticipate that the substantial sample size here could partially compensate for the lack of population diversity. |
| 16 | **Interpretation** |  |  |  |
|  | a) | Meaning: Give a cautious overall interpretation of results in the context of their limitations and in comparison, with other studies | 14 | In our two-sample bidirectional UVMR analyses, we found an association between GGT and CES risk; however, this association was attenuated in MVMR analyses. The discrepancy with previous findings may be attributed to our more stringent selection of covariates. |
|  | b) | Mechanism: Discuss underlying biological mechanisms that could drive a potential causal relationship between the investigated exposure and the outcome, and whether the gene-environment equivalence assumption is reasonable. Use causal language carefully, clarifying that IV estimates may provide causal effects only under certain assumptions | 13-15 | As a regulator of glutathione metabolism, GGT plays a crucial role in maintaining antioxidant homeostasis by recycling extracellular glutathione, which is essential for metabolic processes. Elevated serum GGT levels are indicative of enhanced inflammatory states and increased oxidative stress. Consequently, GGT may accelerate atherosclerosis progression via oxidative and inflammatory mechanisms. Additionally, several studies have reported an association between GGT and atrial fibrillation, attributed to oxidative stress, chronic low-grade inflammation, and metabolic syndrome. This suggests a potential overlap between elevated GGT levels and the underlying mechanisms of stroke occurrence, which could help elucidate how elevated GGT contributes to an increased stroke risk. In our stratified analyses, sex disparities were observed in the correlation between GGT levels and HS risk, particularly indicating a correlation with ICH risk in males rather than females. Several factors may contribute to this observation. First, ICH is more prevalent in males. Second, a combination of genetic and environmental factors substantially influences the biological effects of liver enzyme levels, encompassing metabolic risk factors, inflammatory markers, alcohol consumption, smoking habits, and coffee intake.  Increased bone metabolism activity and the promotion of vascular homeostasis are mechanisms that may elucidate why elevated ALP levels could pose a risk factor for stroke, particularly IS. Previous studies indicates that serum ALP levels typically elevate with age, especially among postmenopausal women. Therefore, the sex differences observed in our findings might be linked to several factors contributing to heightened ALP levels, including age, female, bone disorders, chronic kidney disease (CKD), and inflammation. Interestingly, females exhibited a higher incidence of SAH but a lower incidence of IS.  Elevated serum ALT has been identified as an independent marker of systemic inflammation and increased oxidative stress, which may contribute to vascular fragility and the occurrence of hemorrhagic events. |
|  | c) | Clinical relevance: Discuss whether the results have clinical or public policy relevance, and to what extent they inform effect sizes of possible interventions | 16 | Finally, and most importantly, this study offers notable public health and clinical guidance. Previous studies have mostly focused on exploring the potential biomarker of ALP as prognostic tools, noting an association between elevated ALP levels and increased mortality in stroke patients, or identifying a correlation between ALP and stroke incidence among high-risk individuals. However, our study underscores the prospective role of ALP as a predictive biomarker for stroke risk in the general population, which could assist clinicians in identifying and intervening in high-risk groups. |
| 17 | **Generalizability** | Discuss the generalizability of the study results (a) to other populations, (b) across other exposure periods/timings, and (c) across other levels of exposure | 16 | (3) Lack of sex-specific causal evidence is due to limitations in stroke GWAS data stratified by sex. (4) MR analyses are restricted to European population, potentially limiting the generalizability of the study findings. |
|  | **OTHER INFORMATION** |  |  |  |
| 18 | **Funding** | Describe sources of funding and the role of funders in the present study and, if applicable, sources of funding for the databases and original study or studies on which the present study is based | 17 | This work was supported by grants from the National Natural Science Foundation of China (82171555), Central Nervous System Drug Key Laboratory of Sichuan Province (230003-01SZ), Development and Application of Human Major Disease Monkey Model Key Laboratory of Sichuan Province (2023KF002), and Luzhou Science and Technology Program (2022SWMU4). |
| 19 | **Data and data sharing** | Provide the data used to perform all analyses or report where and how the data can be accessed, and reference these sources in the article. Provide the statistical code needed to reproduce the results in the article, or report whether the code is publicly accessible and if so, where | 18 | Datasets for GWAS summary statistics on liver enzymes are publicly accessible via the NHGRI-EBI GWAS Catalog (https://www.ebi.ac.uk/gwas/), with accession codes GCST90013405-GCST90013407 and GSCT90025980. GWAS summary statistics for stroke, IS and its subtypes, can be found in the NHGRI-EBI GWAS Catalog under accession codes GCST90104539-GCST90104543. GWAS summary statistics for SAH and ICH are accessible through the FinnGen R10 release (https://www.finngen.fi/en/access_results), with phenocodes I9_SAH and I9_INTRACRA, respectively. Other GWAS data sources are detailed in Table S2. |
| 20 | **Conflicts of Interest** | All authors should declare all potential conflicts of interest | 18 | All authors declare no competing interests. |

This checklist is copyrighted by the Equator Network under the Creative Commons Attribution 3.0 Unported (CC BY 3.0) license.

1. Skrivankova VW, Richmond RC, Woolf BAR, Yarmolinsky J, Davies NM, Swanson SA, et al. Strengthening the Reporting of Observational Studies in Epidemiology using Mendelian Randomization (STROBE-MR) Statement. JAMA. 2021;under review.

2. Skrivankova VW, Richmond RC, Woolf BAR, Davies NM, Swanson SA, VanderWeele TJ, et al. Strengthening the Reporting of Observational Studies in Epidemiology using Mendelian Randomisation (STROBE-MR): Explanation and Elaboration. BMJ. 2021;375:n2233.
