# Supplementary material for: Serum liver enzymes and risk of stroke: Systematic review with meta‐analyses and Mendelian randomization studies
Source: Eur J Neurol. 2024 Oct 10;31(12):e16506. doi: 10.1111/ene.16506 (PMC11555028; doi:10.1111/ene.16506)
Supplement: Supplementary file 3 — Supporting Information File 3. [file ENE-31-e16506-s001.docx]

**Additional file 3:**

**3.1 Mate-analyses retrieval record**

**PUBMED 2024.02.26**

| **Search number** | **Query** | **Results** |
| --- | --- | --- |
| 1 | (Alkaline Phosphatase[MeSH Terms]) OR (Alkaline Phosphatase[Title/Abstract]) | 103297 |
| 2 | (((((((((((((((((((((Aspartate Aminotransferases[MeSH Terms]) OR (Aminotransferases, Aspartate[Title/Abstract])) OR (Aspartate Apoaminotransferase[Title/Abstract])) OR (Apoaminotransferase, Aspartate[Title/Abstract])) OR (Aspartate Transaminase[Title/Abstract])) OR (Transaminase, Aspartate[Title/Abstract])) OR (Glutamic-Oxaloacetic Transaminase[Title/Abstract])) OR (Glutamic Oxaloacetic Transaminase[Title/Abstract])) OR (Transaminase, Glutamic-Oxaloacetic[Title/Abstract])) OR (L-Aspartate-2-Oxoglutarate Aminotransferase[Title/Abstract])) OR (Aminotransferase, L-Aspartate-2-Oxoglutarate[Title/Abstract])) OR (L Aspartate 2 Oxoglutarate Aminotransferase[Title/Abstract])) OR (Aspartate Aminotransferase[Title/Abstract])) OR (Aminotransferase, Aspartate[Title/Abstract])) OR (Glutamate-Aspartate Transaminase[Title/Abstract])) OR (Glutamate Aspartate Transaminase[Title/Abstract])) OR (Transaminase, Glutamate-Aspartate[Title/Abstract])) OR (SGOT[Title/Abstract])) OR (Serum Glutamic-Oxaloacetic Transaminase[Title/Abstract])) OR (Glutamic-Oxaloacetic Transaminase, Serum[Title/Abstract])) OR (Serum Glutamic Oxaloacetic Transaminase[Title/Abstract])) OR (Transaminase, Serum Glutamic-Oxaloacetic[Title/Abstract]) | 57916 |
| 3 | (((((((((((((Alanine Transaminase[MeSH Terms]) OR (Transaminase, Alanine[Title/Abstract])) OR (Glutamic-Alanine Transaminase[Title/Abstract])) OR (Glutamic Alanine Transaminase[Title/Abstract])) OR (Transaminase, Glutamic-Alanine[Title/Abstract])) OR (Alanine-2-Oxoglutarate Aminotransferase[Title/Abstract])) OR (Alanine 2 Oxoglutarate Aminotransferase[Title/Abstract])) OR (Aminotransferase, Alanine-2-Oxoglutarate[Title/Abstract])) OR (Alanine Aminotransferase[Title/Abstract])) OR (Aminotransferase, Alanine[Title/Abstract])) OR (Glutamic-Pyruvic Transaminase[Title/Abstract])) OR (Glutamic Pyruvic Transaminase[Title/Abstract])) OR (Transaminase, Glutamic-Pyruvic[Title/Abstract])) OR (SGPT[Title/Abstract]) | 60288 |
| 4 | ((((((((gamma Glutamyltransferase[MeSH Terms]) OR (gamma Glutamyltransferase[Title/Abstract])) OR (GGTP[Title/Abstract])) OR (gammaglutamyltransferase[Title/Abstract])) OR (Glutamyl Transpeptidase[Title/Abstract])) OR (Transpeptidase, Glutamyl[Title/Abstract])) OR (gamma-Glutamyl Transpeptidase[Title/Abstract])) OR (Transpeptidase, gamma-Glutamyl[Title/Abstract])) OR (gamma Glutamyl Transpeptidase[Title/Abstract]) | 18378 |
| 5 | #1 OR #2 OR #3 OR #4 | 183479 |
| 6 | ((((((((((((((((((((((((((((Stroke[MeSH Terms]) OR (Strokes[Title/Abstract])) OR (Cerebrovascular Accident[Title/Abstract])) OR (Cerebrovascular Accidents[Title/Abstract])) OR (CVA (Cerebrovascular Accident[Title/Abstract]))) OR (CVAs (Cerebrovascular Accident[Title/Abstract]))) OR (Cerebrovascular Apoplexy[Title/Abstract])) OR (Apoplexy, Cerebrovascular[Title/Abstract])) OR (Vascular Accident, Brain[Title/Abstract])) OR (Brain Vascular Accident[Title/Abstract])) OR (Brain Vascular Accidents[Title/Abstract])) OR (Vascular Accidents, Brain[Title/Abstract])) OR (Cerebrovascular Stroke[Title/Abstract])) OR (Cerebrovascular Strokes[Title/Abstract])) OR (Stroke, Cerebrovascular[Title/Abstract])) OR (Strokes, Cerebrovascular[Title/Abstract])) OR (Apoplexy[Title/Abstract])) OR (Cerebral Stroke[Title/Abstract])) OR (Cerebral Strokes[Title/Abstract])) OR (Stroke, Cerebral[Title/Abstract])) OR (Strokes, Cerebral[Title/Abstract])) OR (Stroke, Acute[Title/Abstract])) OR (Acute Stroke[Title/Abstract])) OR (Acute Strokes[Title/Abstract])) OR (Strokes, Acute[Title/Abstract])) OR (Cerebrovascular Accident, Acute[Title/Abstract])) OR (Acute Cerebrovascular Accident[Title/Abstract])) OR (Acute Cerebrovascular Accidents[Title/Abstract])) OR (Cerebrovascular Accidents, Acute[Title/Abstract]) | 209557 |
| 7 | (((((((((((((((((((((((Prospective Studies [MeSH Terms]) OR (Prospective Study)) OR (Studies, Prospective)) OR (Study, Prospective)) OR (Follow-Up Studies[MeSH Terms])) OR (Follow Up Studies)) OR (Follow-Up Study)) OR (Studies, Follow-Up)) OR (Study, Follow-Up)) OR (Followup Studies)) OR (Followup Study)) OR (Studies, Followup)) OR (Study, Followup)) OR (Nested Case Control Studies)) OR (Nested Case-Control Studies)) OR (Case-Control Studies, Nested)) OR (Case-Control Study, Nested)) OR (Nested Case-Control Study)) OR (Studies, Nested Case-Control)) OR (Study, Nested Case-Control)) OR (Real World Study)) OR (Real World Research)) OR (Real World Data)) OR (Real World Evidence) | 3271280 |
| 8 | ((((((Humans [MeSH Terms])) OR (Homo sapiens)) OR (Man (Taxonomy))) OR (Man, Modern)) OR (Modern Man)) OR (Human) | 22,689,050 |
| 9 | #5 AND #6 AND #7 AND #8 | 108 |

**EMBASE 2024.02.26**

| **Search number** | **Query** | **Results** |
| --- | --- | --- |
| 1 | 'cerebrovascular accident'/exp OR 'cerebrovascular accident' | 448886 |
| 2 | 'Strokes':ab,ti or 'Cerebrovascular Accident':ab,ti or 'Cerebrovascular Accidents':ab,ti or 'CVA (Cerebrovascular Accident)':ab,ti or 'CVAs (Cerebrovascular Accident)':ab,ti or 'Cerebrovascular Apoplexy':ab,ti or 'Apoplexy, Cerebrovascular':ab,ti or 'Vascular Accident, Brain':ab,ti or 'Brain Vascular Accident':ab,ti or 'Brain Vascular Accidents':ab,ti or 'Vascular Accidents, Brain':ab,ti or 'Cerebrovascular Stroke':ab,ti or 'Cerebrovascular Strokes':ab,ti or 'Stroke, Cerebrovascular':ab,ti or 'Strokes, Cerebrovascular':ab,ti or 'Apoplexy':ab,ti or 'Cerebral Stroke':ab,ti or 'Cerebral Strokes':ab,ti or 'Stroke, Cerebral':ab,ti or 'Strokes, Cerebral':ab,ti or 'Stroke, Acute':ab,ti or 'Acute Stroke':ab,ti or 'Acute Strokes':ab,ti or 'Strokes, Acute':ab,ti or 'Cerebrovascular Accident, Acute':ab,ti or 'Acute Cerebrovascular Accident':ab,ti or 'Acute Cerebrovascular Accidents':ab,ti or 'Cerebrovascular Accidents, Acute':ab,ti | 99193 |
| 3 | #1 OR #2 | 477189 |
| 4 | 'alkaline phosphatase'/exp OR 'alkaline phosphatase' | 184410 |
| 5 | 'aspartate aminotransferase'/exp OR 'aspartate aminotransferase' | 184026 |
| 6 | 'Aminotransferases, Aspartate':ab,ti or 'Aspartate Apoaminotransferase':ab,ti or 'Apoaminotransferase, Aspartate':ab,ti or 'Aspartate Transaminase':ab,ti or 'Transaminase, Aspartate':ab,ti or 'Glutamic-Oxaloacetic Transaminase':ab,ti or 'Glutamic Oxaloacetic Transaminase':ab,ti or 'Transaminase, Glutamic-Oxaloacetic':ab,ti or 'L-Aspartate-2-Oxoglutarate Aminotransferase':ab,ti or 'Aminotransferase, L-Aspartate-2-Oxoglutarate':ab,ti or 'L Aspartate 2 Oxoglutarate Aminotransferase':ab,ti or 'Aspartate Aminotransferase':ab,ti or 'Aminotransferase, Aspartate':ab,ti or 'Glutamate-Aspartate Transaminase':ab,ti or 'Glutamate Aspartate Transaminase':ab,ti or 'Transaminase, Glutamate-Aspartate':ab,ti or 'SGOT':ab,ti or 'Serum Glutamic-Oxaloacetic Transaminase':ab,ti or 'Glutamic-Oxaloacetic Transaminase, Serum':ab,ti or 'Serum Glutamic Oxaloacetic Transaminase':ab,ti or 'Transaminase, Serum Glutamic-Oxaloacetic':ab,ti | 52106 |
| 7 | 'alanine aminotransferase'/exp OR 'alanine aminotransferase' | 199431 |
| 8 | 'Transaminase, Alanine':ab,ti or 'Glutamic-Alanine Transaminase':ab,ti or 'Glutamic Alanine Transaminase':ab,ti or 'Transaminase, Glutamic-Alanine':ab,ti or 'Alanine-2-Oxoglutarate Aminotransferase':ab,ti or 'Alanine 2 Oxoglutarate Aminotransferase':ab,ti or 'Aminotransferase, Alanine-2-Oxoglutarate':ab,ti or 'Alanine Aminotransferase':ab,ti or 'Aminotransferase, Alanine':ab,ti or 'Glutamic-Pyruvic Transaminase':ab,ti or 'Glutamic Pyruvic Transaminase':ab,ti or 'Transaminase, Glutamic-Pyruvic':ab,ti or 'SGPT':ab,ti | 54561 |
| 9 | 'gamma glutamyltransferase'/exp OR 'gamma glutamyltransferase' | 67916 |
| 10 | 'gamma Glutamyltransferase':ab,ti or 'GGTP':ab,ti or 'gammaglutamyltransferase':ab,ti or 'Glutamyl Transpeptidase':ab,ti or 'Transpeptidase, Glutamyl':ab,ti or 'gamma-Glutamyl Transpeptidase':ab,ti or 'Transpeptidase, gamma-Glutamyl':ab,ti or 'gamma Glutamyl Transpeptidase':ab,ti | 12214 |
| 11 | #4 OR #5 OR #6 OR #7 OR #8 OR #9 OR #10 | 408544 |
| 12 | #3 AND #11 | 3073 |
| 13 | 'prospective study'/exp OR 'prospective study' | 971136 |
| 14 | 'Prospective Studies':ab,ti or 'Studies, Prospective':ab,ti or 'Study, Prospective':ab,ti | 79989 |
| 15 | 'Follow-Up Studies':ab,ti or 'Follow Up Studies':ab,ti or 'Follow-Up Study':ab,ti or 'Studies, Follow-Up':ab,ti or 'Study, Follow-Up':ab,ti or 'Followup Studies':ab,ti or 'Followup Study':ab,ti or 'Studies, Followup':ab,ti or 'Study, Followup':ab,ti or 'Nested Case Control Studies':ab,ti or 'Nested Case-Control Studies':ab,ti or 'Case-Control Studies, Nested':ab,ti or 'Case-Control Study, Nested':ab,ti or 'Nested Case-Control Study':ab,ti or 'Studies, Nested Case-Control':ab,ti or 'Study, Nested Case-Control':ab,ti or 'Real World Study':ab,ti or 'Real World Research':ab,ti or 'Real World Data':ab,ti or 'Real World Evidence':ab,ti | 131530 |
| 16 | #13 OR #14 OR #15 | 1124959 |
| 17 | 'human'/exp OR 'human' | 28,379,615 |
| 18 | 'Humans':ab,ti or 'Homo sapiens':ab,ti or 'Man (Taxonomy)':ab,ti or 'Man, Modern':ab,ti or 'Modern Man':ab,ti | 575203 |
| 19 | #17 OR #18 | 28,496,980 |
| 20 | #12 AND #16 AND #19 | 345 |

**SCOPUS 2024.02.26**

| **Search number** | **Query** | **Results** |
| --- | --- | --- |
| 1 | "Stroke" or "Strokes" or "Cerebrovascular Accident" or "Cerebrovascular Accidents" or "CVA (Cerebrovascular Accident)" or "CVAs (Cerebrovascular Accident)" or "Cerebrovascular Apoplexy" or "Apoplexy, Cerebrovascular" or "Vascular Accident, Brain" or "Brain Vascular Accident" or "Brain Vascular Accidents" or "Vascular Accidents, Brain" or "Cerebrovascular Stroke" or "Cerebrovascular Strokes" or "Stroke, Cerebrovascular" or "Strokes, Cerebrovascular" or "Apoplexy" or "Cerebral Stroke" or "Cerebral Strokes" or "Stroke, Cerebral" or "Strokes, Cerebral" or "Stroke, Acute" or "Acute Stroke" or "Acute Strokes" or "Strokes, Acute" or "Cerebrovascular Accident, Acute" or "Acute Cerebrovascular Accident" or "Acute Cerebrovascular Accidents" or "Cerebrovascular Accidents, Acute" | 618975 |
| 2 | "Alkaline Phosphatase" or "Aspartate Aminotransferases" or "Aminotransferases, Aspartate" or "Aspartate Apoaminotransferase" or "Apoaminotransferase, Aspartate" or "Aspartate Transaminase" or "Transaminase, Aspartate" or "Glutamic-Oxaloacetic Transaminase" or "Glutamic Oxaloacetic Transaminase" or "Transaminase, Glutamic-Oxaloacetic" or "L-Aspartate-2-Oxoglutarate Aminotransferase" or "Aminotransferase, L-Aspartate-2-Oxoglutarate" or "L Aspartate 2 Oxoglutarate Aminotransferase" or "Aspartate Aminotransferase" or "Aminotransferase, Aspartate" or "Glutamate-Aspartate Transaminase" or "Glutamate Aspartate Transaminase" or "Transaminase, Glutamate-Aspartate" or "SGOT" or "Serum Glutamic-Oxaloacetic Transaminase" or "Glutamic-Oxaloacetic Transaminase, Serum" or "Serum Glutamic Oxaloacetic Transaminase" or "Transaminase, Serum Glutamic-Oxaloacetic" or "Alanine Transaminase" or "Transaminase, Alanine" or "Glutamic-Alanine Transaminase" or "Glutamic Alanine Transaminase" or "Transaminase, Glutamic-Alanine" or "Alanine-2-Oxoglutarate Aminotransferase" or "Alanine 2 Oxoglutarate Aminotransferase" or "Aminotransferase, Alanine-2-Oxoglutarate" or "Alanine Aminotransferase" or "Aminotransferase, Alanine" or "Glutamic-Pyruvic Transaminase" or "Glutamic Pyruvic Transaminase" or "Transaminase, Glutamic-Pyruvic" or "SGPT" or "gamma Glutamyltransferase" or "gamma Glutamyltransferase" or "GGTP" or "gammaglutamyltransferase" or "Glutamyl Transpeptidase" or "Transpeptidase, Glutamyl" or "gamma-Glutamyl Transpeptidase" or "Transpeptidase, gamma-Glutamyl" or "gamma Glutamyl Transpeptidase" | 386,935 |
| 3 | "Prospective Studies" or "Prospective Study" or "Studies, Prospective" or "Study, Prospective" or "Follow-Up Studies " or "Follow Up Studies" or "Follow-Up Study" or "Studies, Follow-Up" or "Study, Follow-Up" or "Followup Studies" or "Followup Study" or "Studies, Followup" or "Study, Followup" or "Nested Case Control Studies" or "Nested Case-Control Studies" or "Case-Control Studies, Nested" or "Case-Control Study, Nested" or "Nested Case-Control Study" or "Studies, Nested Case-Control" or "Study, Nested Case-Control" or "Real World Study" or "Real World Research" or "Real World Data" or "Real World Evidence" | 1,535,941 |
| 4 | "Humans" or "Homo sapiens" or "Man (Taxonomy)" or "Man, Modern" or "Modern Man" or "Human" | 26,751,830 |
| 5 | #1 AND #2 AND #3 AND #4 | 615 |

**3.2 Included studies in meta-analyses^1-17^**

1. Shimizu Y, Imano H, Ohira T, et al. Alkaline phosphatase and risk of stroke among Japanese: the Circulatory Risk in Communities Study (CIRCS). *J Stroke Cerebrovasc Dis*. 2013;22(7):1046-1055.

2. Wannamethee SG, Sattar N, Papcosta O, Lennon L, Whincup PH. Alkaline phosphatase, serum phosphate, and incident cardiovascular disease and total mortality in older men. *Arterioscler Thromb Vasc Biol*. 2013;33(5):1070-1076.

3. Yang Y-J, Jung M-H, Jeong S-H, Hong Y-P, Kim YI, An SJ. The Association between Nonalcoholic Fatty Liver Disease and Stroke: Results from the Korean Genome and Epidemiology Study (KoGES). *International Journal of Environmental Research and Public Health*. 2020;17(24).

4. Liu K, Yu Y, Yuan Y, et al. Elevated Levels of Serum Alkaline Phosphatase are Associated with Increased Risk of Cardiovascular Disease: A Prospective Cohort Study. *Journal of Atherosclerosis and Thrombosis*. 2023;30(7):795-819.

5. Kim HC, Kang DR, Nam CM, et al. Elevated serum aminotransferase level as a predictor of intracerebral hemorrhage: Korea medical insurance corporation study. *Stroke*. 2005;36(8):1642-1647.

6. Bots ML, Salonen JT, Elwood PC, et al. Gamma-glutamyltransferase and risk of stroke: the EUROSTROKE project. *J Epidemiol Community Health*. 2002;56 Suppl 1(Suppl 1):i25-i29.

7. Fraser A, Harris R, Sattar N, Ebrahim S, Smith GD, Lawlor DA. Gamma-glutamyltransferase is associated with incident vascular events independently of alcohol intake: analysis of the British Women's Heart and Health Study and Meta-Analysis. *Arterioscler Thromb Vasc Biol*. 2007;27(12):2729-2735.

8. Shimizu Y, Imano H, Ohira T, et al. gamma-Glutamyltranspeptidase and incident stroke among Japanese men and women: the Circulatory Risk in Communities Study (CIRCS). *Stroke*. 2010;41(2):385-388.

9. Wang X, Cheng S, Lv J, et al. Liver biomarkers, genetic and lifestyle risk factors in relation to risk of cardiovascular disease in Chinese. *Front Cardiovasc Med*. 2022;9:938902.

10. Weikert C, Drogan D, di Giuseppe R, et al. Liver enzymes and stroke risk in middle-aged German adults. *Atherosclerosis*. 2013;228(2):508-514.

11. Arafa A, Kokubo Y, Kashima R, Matsumoto C, Koga M. Liver enzymes and the risk of stroke among the general Japanese population: a prospective cohort study. *Cerebrovascular Diseases (Basel, Switzerland)*. 2023.

12. Baek H-S, Kim B, Lee S-H, et al. Long-Term Cumulative Exposure to High γ-Glutamyl Transferase Levels and the Risk of Cardiovascular Disease: A Nationwide Population-Based Cohort Study. *Endocrinol Metab (Seoul)*. 2023;38(6):770-781.

13. Kabootari M, Raee MR, Akbarpour S, Asgari S, Azizi F, Hadaegh F. Serum alkaline phosphatase and the risk of coronary heart disease, stroke and all-cause mortality: Tehran Lipid and Glucose Study. *BMJ Open*. 2018;8(11):e023735.

14. Ebrahim S, Sung J, Song Y-M, Ferrer RL, Lawlor DA, Davey Smith G. Serum cholesterol, haemorrhagic stroke, ischaemic stroke, and myocardial infarction: Korean national health system prospective cohort study. *BMJ*. 2006;333(7557):22.

15. Jousilahti P, Rastenyte D, Tuomilehto J. Serum gamma-glutamyl transferase, self-reported alcohol drinking, and the risk of stroke. *Stroke*. 2000;31(8):1851-1855.

16. R.G. Wieberdink PJK, A. Hofman, M.M.B. Breteler, M.A. Ikram. Serum liver enzymes and the risk of stroke in the general population: The rotterdam study. *20th European Stroke Conference, ESC 2011 Hamburg Germany*. 2011;(Erasmus University Medical Center, Rotterdam, The Netherlands)

17. Wannamethee SG, Lennon L, Shaper AG. The value of gamma-glutamyltransferase in cardiovascular risk prediction in men without diagnosed cardiovascular disease or diabetes. *Atherosclerosis*. 2008;201(1):168-175.
