# Supplementary material for: Serum liver enzymes and risk of stroke: Systematic review with meta‐analyses and Mendelian randomization studies
Source: Eur J Neurol. 2024 Oct 10;31(12):e16506. doi: 10.1111/ene.16506 (PMC11555028; doi:10.1111/ene.16506)
Supplement: Supplementary file 5 — Supporting Information File 5. [file ENE-31-e16506-s004.docx]

**Additional file 5 Figures S1-10:**

**
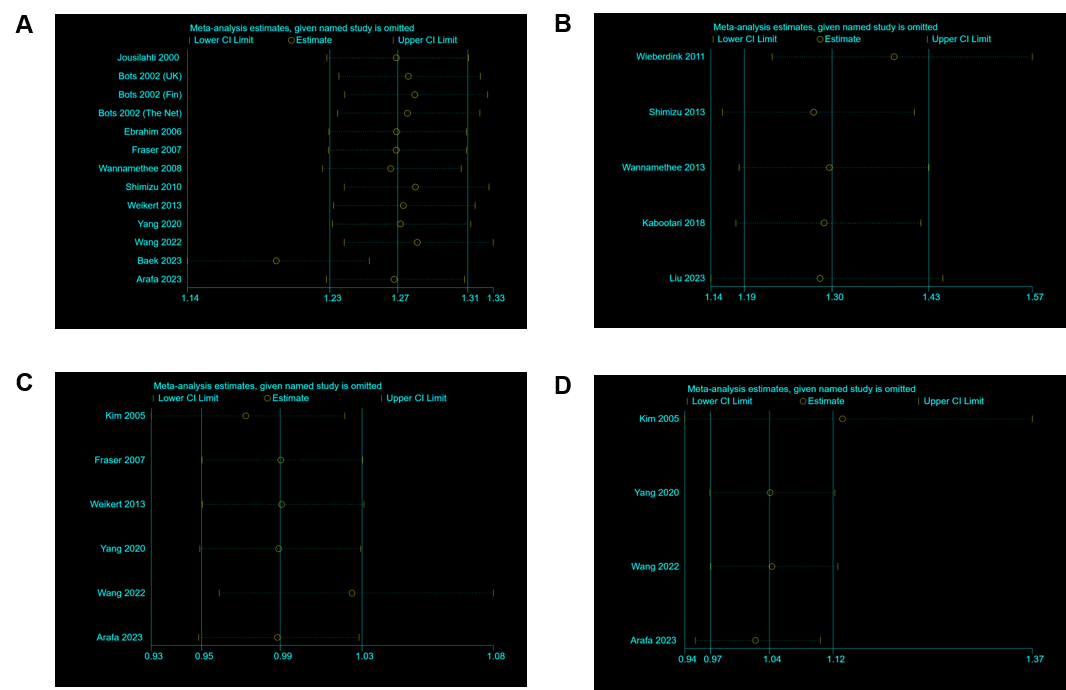
**

**FIGURE S1** The sensitivity analyses of studies on GGT(A), ALP(B), ALT(C), and AST(D). GGT, γ-glutamyl transferase; ALP, alkaline phosphatase; ALT, alanine aminotransferase; AST, aspartate aminotransferase; CI, confidence interval.


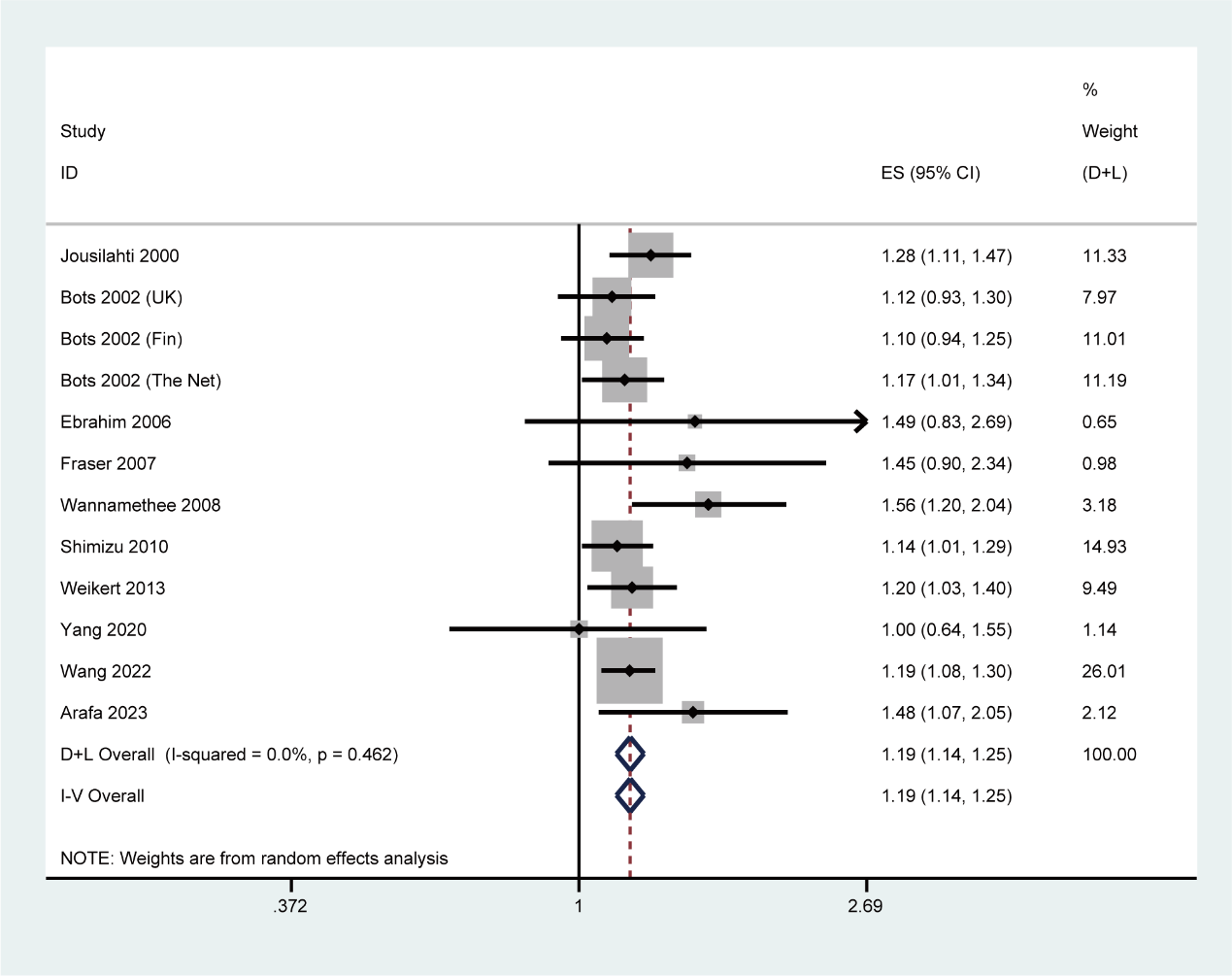


**FIGURE S2** Forest plot of serum GGT (n = 12) and stroke risk post-exclusion of Baek 2023. Dot represents the estimate of relative risk for each study; the size of the box represents the relative weight of the individual study; the horizontal line represents the 95% CI, and the diamond represents the overall estimate and its 95% CI. ES, effect size; CI, confidence interval; D+L, DerSimonian and Laird random effects model; I-V, inverse variance fixed effects model.

**
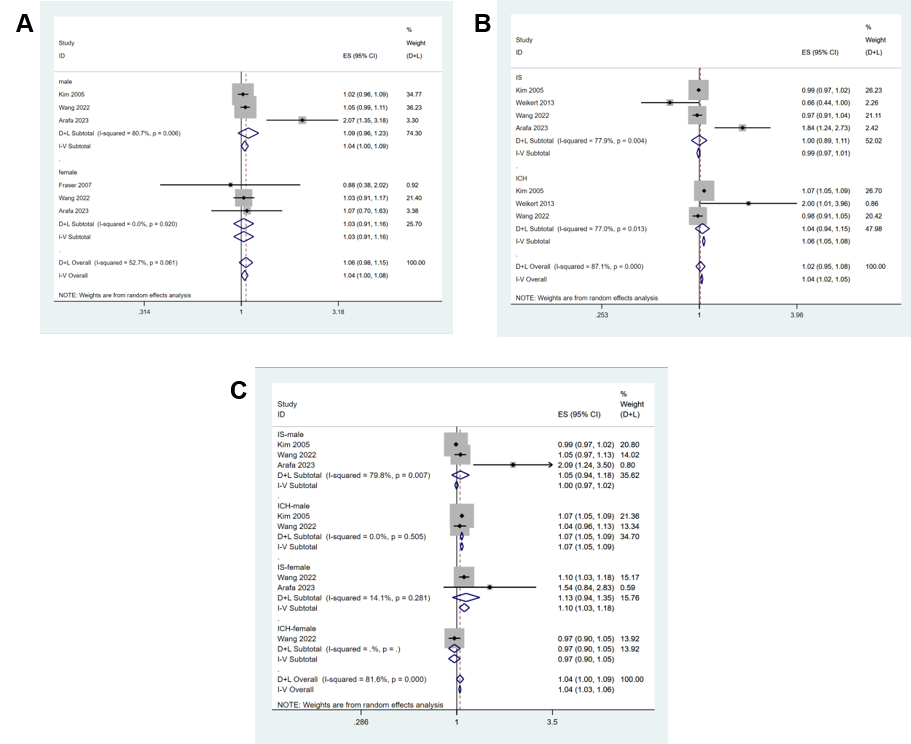
**

**FIGURE S3** Subgroup analyses on the associations of serum ALT levels with stroke stratified by sex (A), stroke subtype (B), and stroke subtype-sex (C). Dot represents the estimate of relative risk for each study; the size of the box represents the relative weight of the individual study; the horizontal line represents the 95% CI, and the diamond represents the overall estimate and its 95% CI. ALT, alanine aminotransferase; IS, ischemic stroke; ICH, intracerebral hemorrhage; ES, effect size; CI, confidence interval; D+L, DerSimonian and Laird random effects model; I-V, inverse variance fixed effects model.


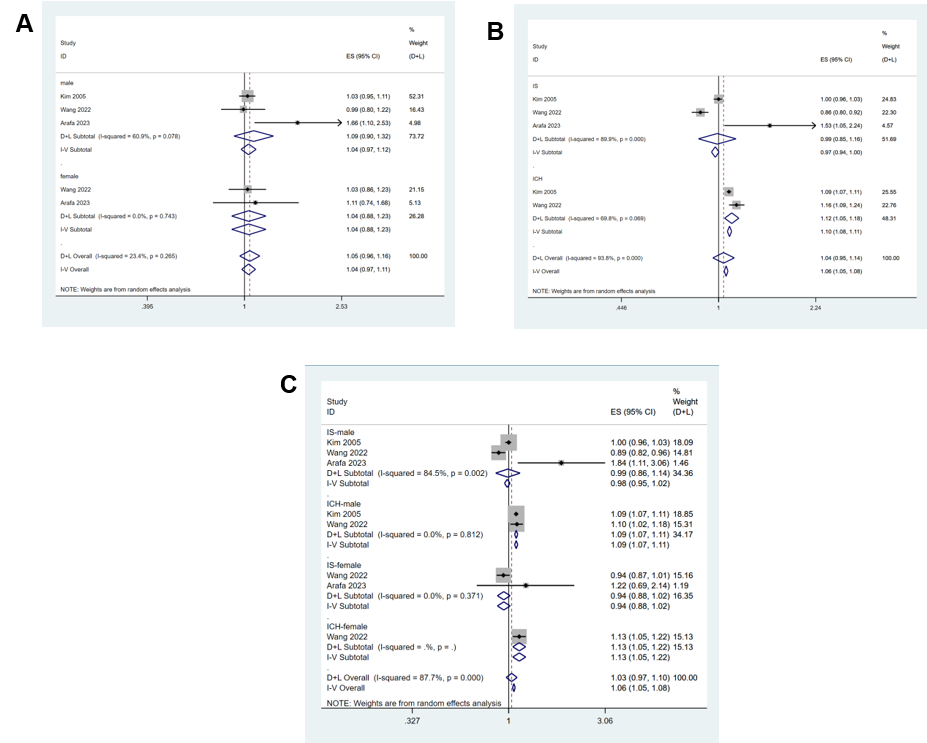


**FIGURE S4** Subgroup analyses on the associations of serum AST levels with stroke stratified by sex (A), stroke subtype (B), and stroke subtype-sex (C). Dot represents the estimate of relative risk for each study; the size of the box represents the relative weight of the individual study; the horizontal line represents the 95% CI, and the diamond represents the overall estimate and its 95% CI. AST, aspartate aminotransferase; IS, ischemic stroke; ICH, intracerebral hemorrhage; ES, effect size; CI, confidence interval; D+L, DerSimonian and Laird random effects model; I-V, inverse variance fixed effects model.

**
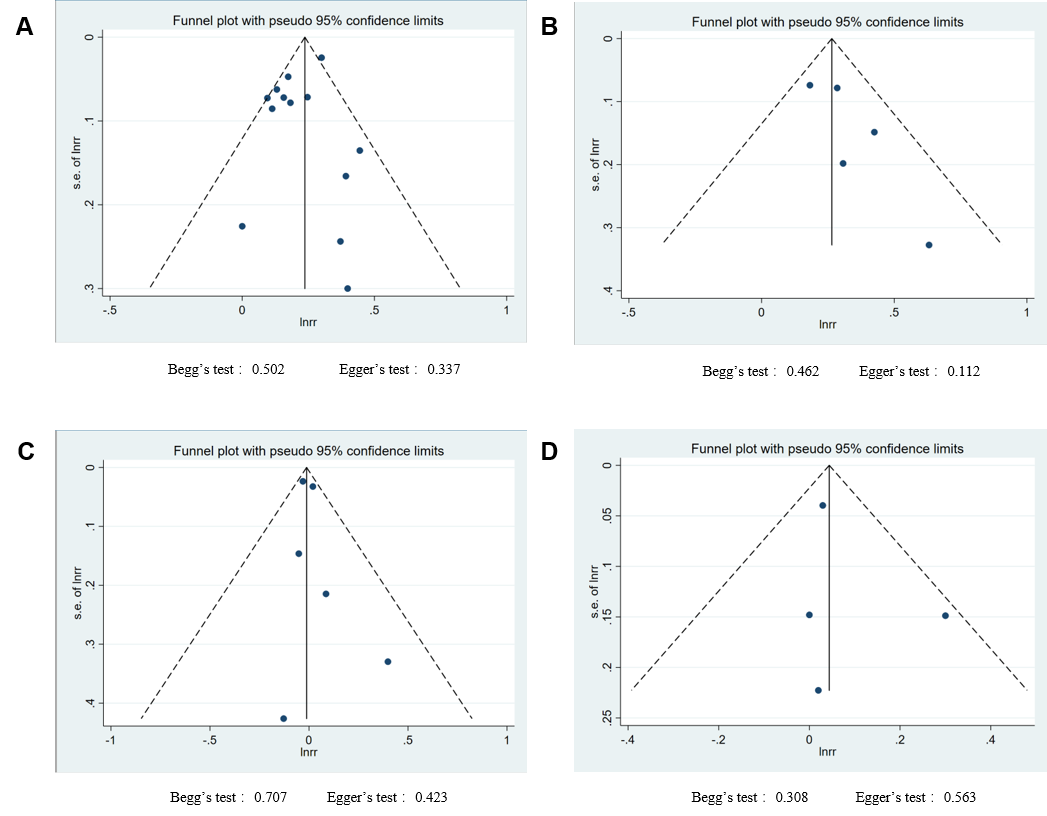
**

**FIGURE S5** Funnel plots and the results of Egger and Begg tests of studies on GGT (A), ALP (B), ALT (C) and AST (D). GGT, γ-glutamyl transferase; ALP, alkaline phosphatase; ALT, alanine aminotransferase; AST, aspartate aminotransferase; RR, relative risk.

**
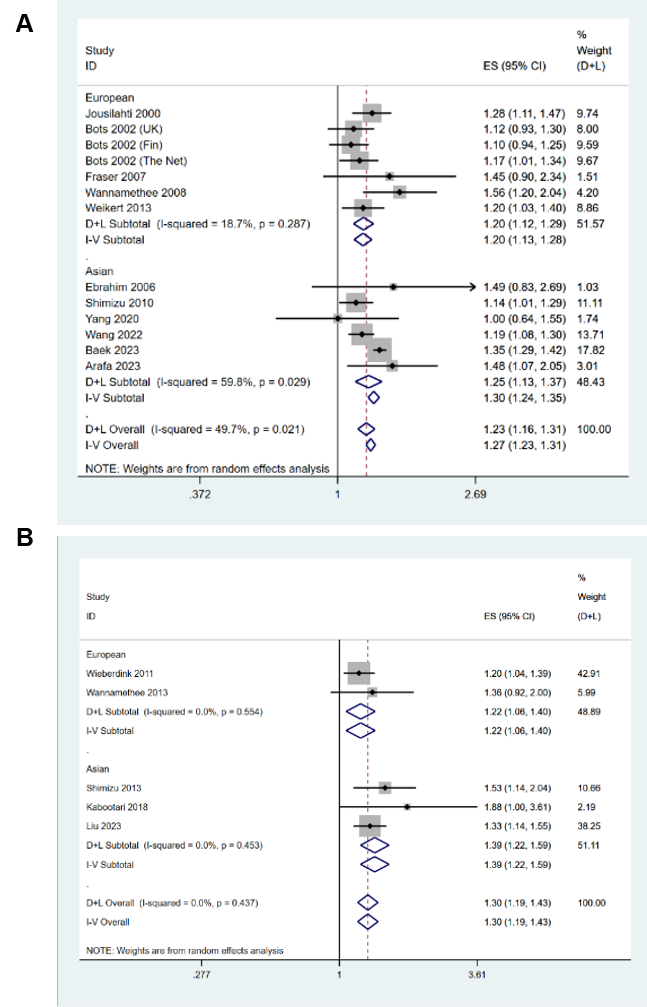
**

**FIGURE S6** Subgroup analyses on the associations of GGT (A) and ALP (B) with stroke risk stratified by population. Dot represents the estimate of relative risk for each study; the size of the box represents the relative weight of the individual study; the horizontal line represents the 95% CI, and the diamond represents the overall estimate and its 95% CI. ES, effect size; CI, confidence interval; D+L, DerSimonian and Laird random effects model; I-V, inverse variance fixed effects model.


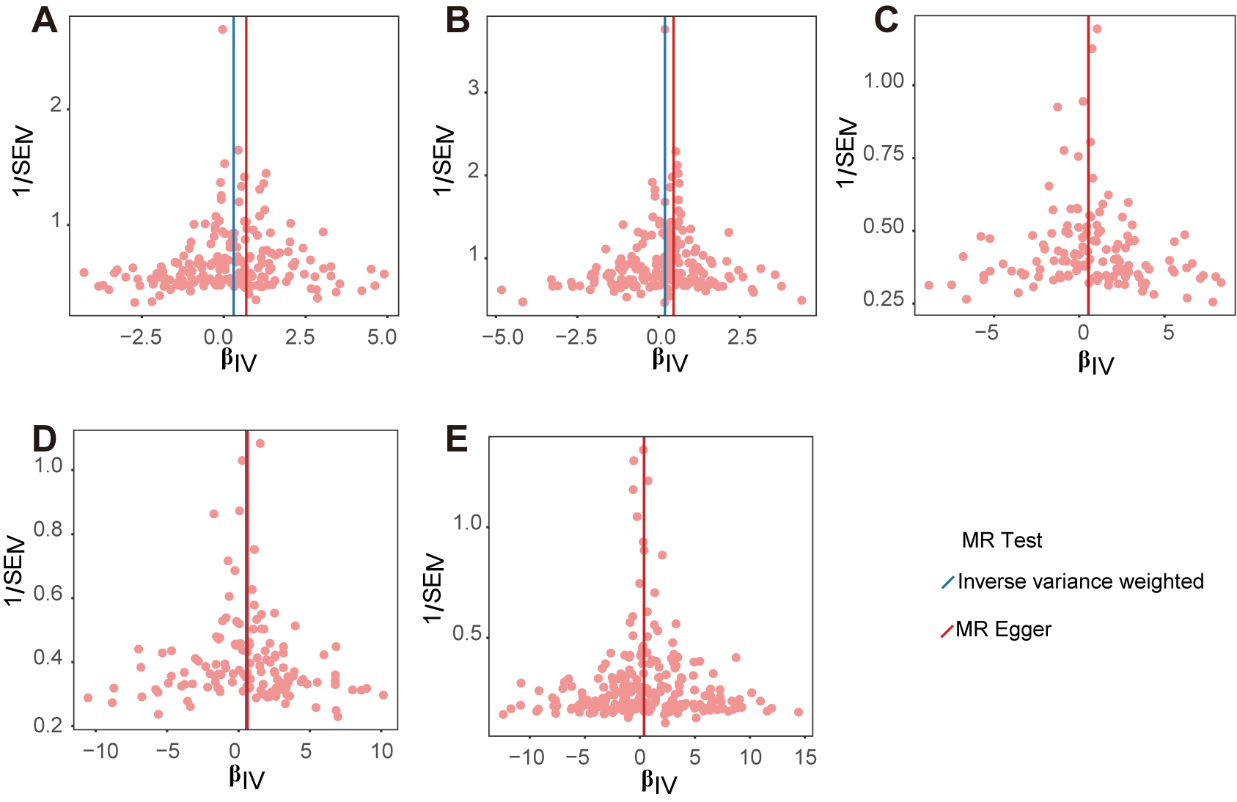


**FIGURE S7** Funnel plots of AST on SAH (A) and ICH (B), ALT on stroke (C) and IS (D), and GGT on CES (E). AST, aspartate aminotransferase; ALT, alanine aminotransferase; GGT, γ-glutamyl transferase; SAH, subarachnoid hemorrhage; ICH, intracerebral hemorrhage; IS, ischemic stroke; CES, cardioembolic stroke; MR, Mendelian randomization.


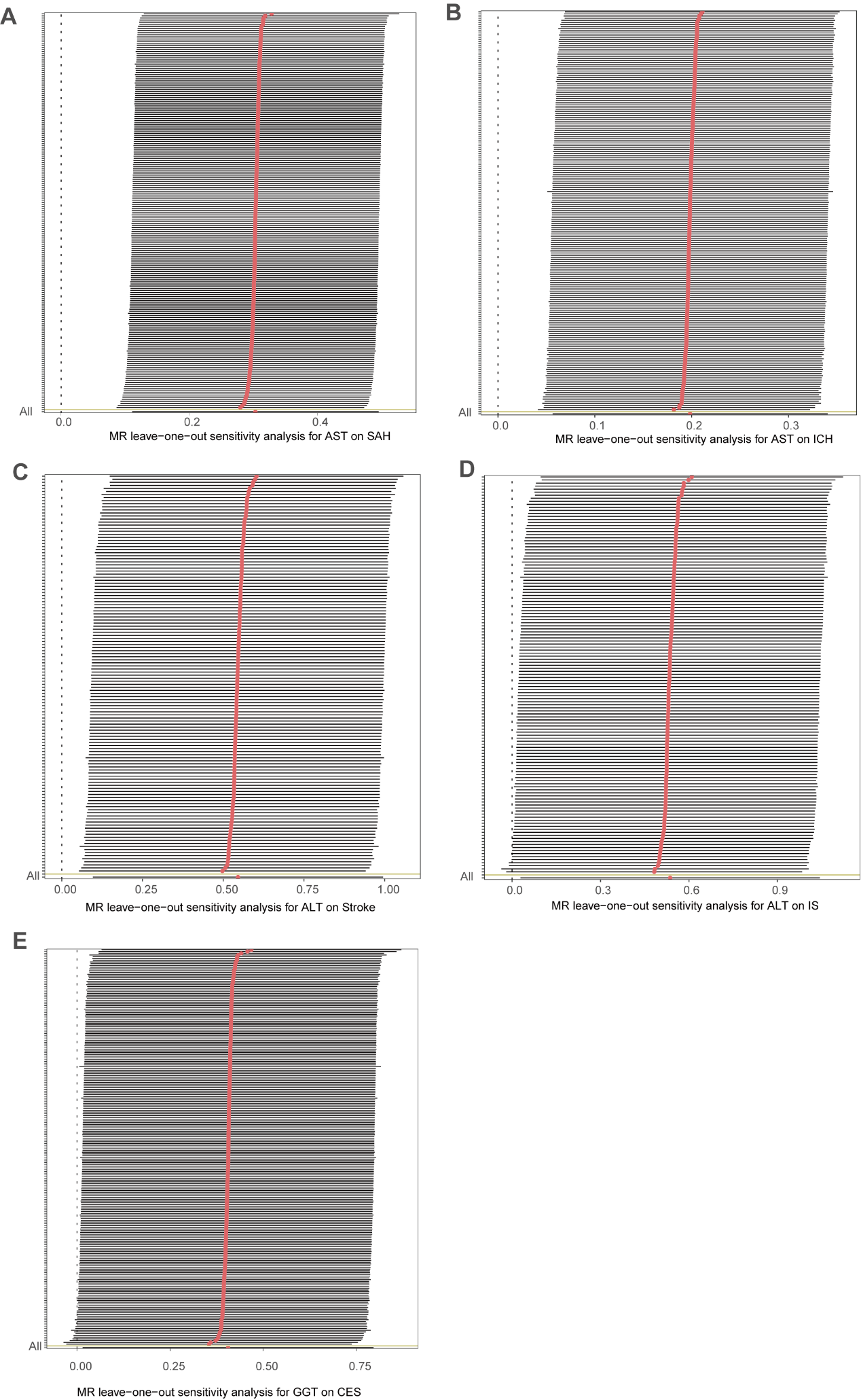


**FIGURE S8** Leave-one-out sensitivity analyses of all significant UVMR results. Leave-one-out sensitivity analyses were conducted to assess causal associations, including AST with SAH (A) and ICH (B), ALT with stroke (C) and IS (D), and GGT with CES (E). Each data point represents the causal estimate when excluding one SNP from the analysis. The x-axis denotes the index of the excluded SNP, while the y-axis represents the effect estimate. AST, aspartate aminotransferase; ALT, alanine aminotransferase; GGT, γ-glutamyl transferase; SAH, subarachnoid hemorrhage; ICH, intracerebral hemorrhage; CES, cardioembolic stroke; MR, Mendelian randomization; UVMR, univariable Mendelian randomization.

**
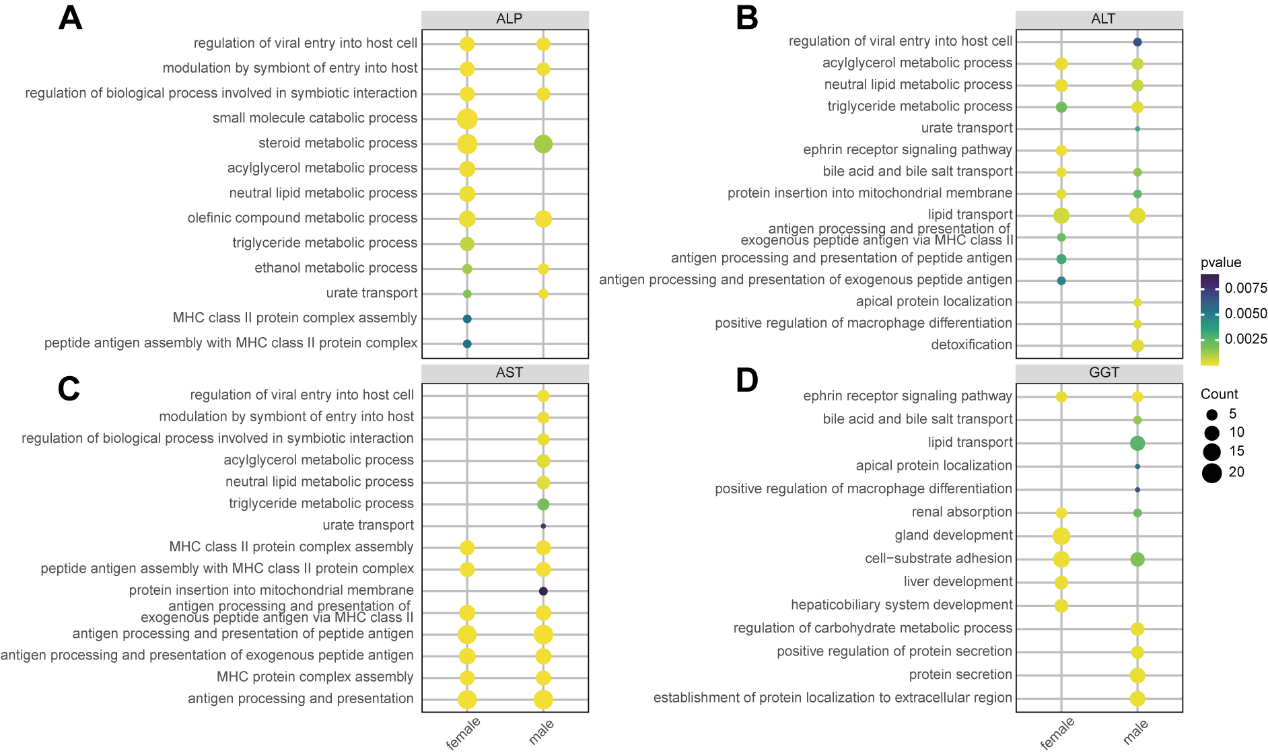
**

**FIGURE S9** Gene set enrichment analyses using genes identified through FUMA for significant associations with liver enzymes across sexes. The x-axis represents sex, the y-axis represents different biological process components of gene ontology. Panels A, B, C, and D correspond to ALP, ALT, AST, and GGT, respectively. Dot size indicates the count of genes, and dot color indicates *p*-value. ALP, alkaline phosphatase; ALT, alanine aminotransferase; AST, aspartate aminotransferase; GGT, γ-glutamyl transferase.

**
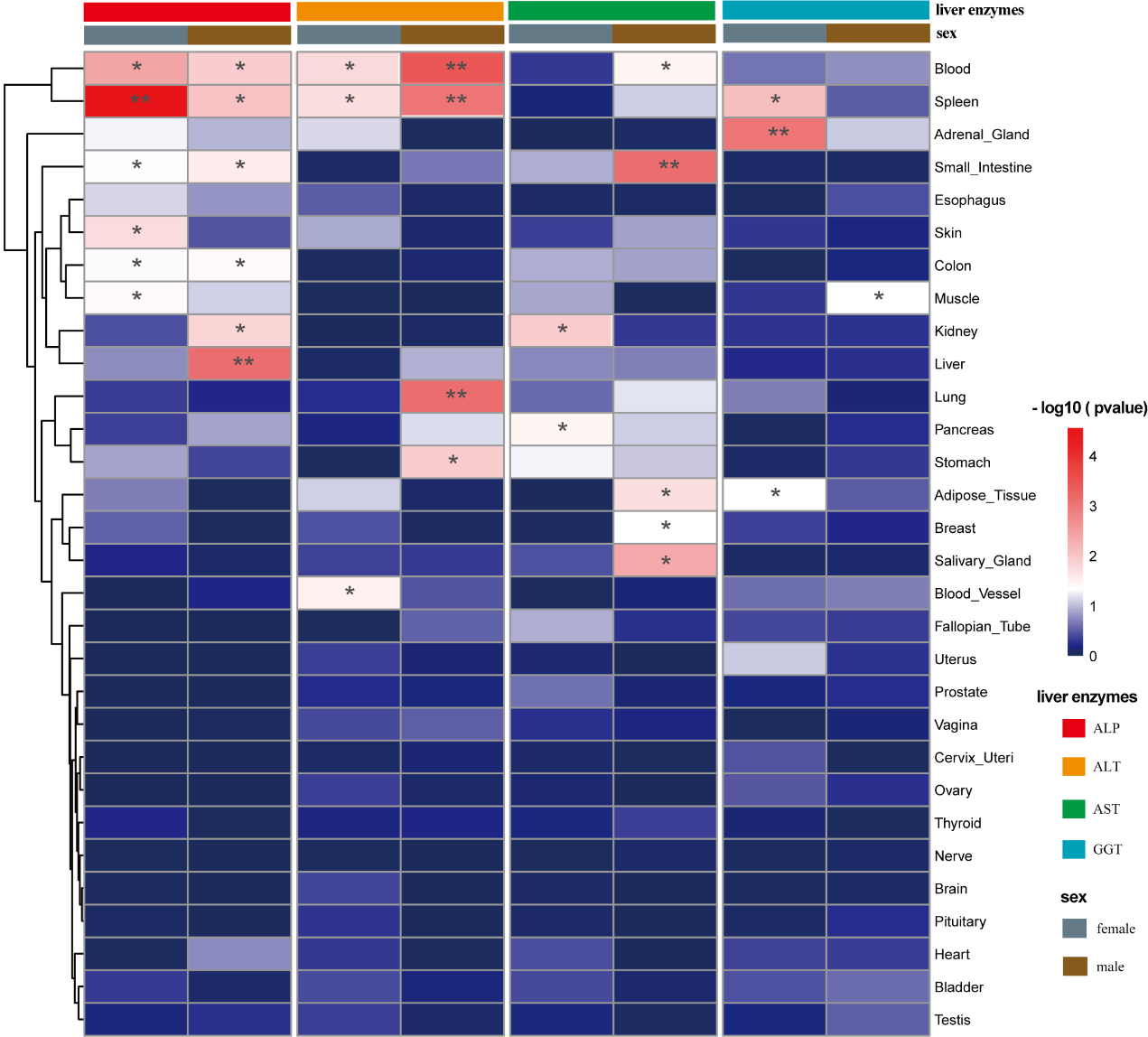
**

**FIGURE S10** Tissue enrichment analyses by FUMA using GTEx v8 for different liver enzymes across sexes. The x-axis represents different liver enzymes and sex, the y-axis represents the 30 general tissues from GTEx v8, and the heatmap color indicates -log10(*p*-values). * indicates nominal significance (*p* < 0.05), and ** indicates statistical significance (*p* < 0.05/30). ALP, alkaline phosphatase; ALT, alanine aminotransferase; AST, aspartate aminotransferase; GGT, γ-glutamyl transferase.
